# Fuzheng Huayu tablets reduces the risk of further decompensation after the first decompensation in patients with HBV-related cirrhosis: protocol for a randomized, double-blind, placebo-controlled, multicenter trial

**DOI:** 10.3389/fphar.2026.1828944

**Published:** 2026-07-02

**Authors:** Lihua Yu, Wenjuan Peng, Wanxin Shi, Heng Ma, Junnan Li, Yong Zhou, Jing Miao, Yixin Hou, Qi Wang, Zhiyun Yang

**Affiliations:** 1 Center of Integrative Medicine, Beijing Ditan Hospital, Capital Medical University, Beijing, China; 2 Science and Technology Department, Beijing Ditan Hospital, Capital Medical University, Beijing, China; 3 Department of Liver Disease, Qingdao Public Health Clinical Center, Qingdao, Shandong, China; 4 Department of Traditional Chinese Medicine, Tianjin Second People’s Hospital, Tianjin, China; 5 Center of Liver Diseases, Beijing Ditan Hospital, Capital Medical University, Beijing, China; 6 Laboratory for Clinical Medicine, Capital Medical University, Beijing, China

**Keywords:** first decompensation, Fuzheng Huayu tablet, HBV-related cirrhosis patients, protocol, randomized controlled trial

## Abstract

**Introduction:**

The first decompensation event of cirrhosis is a critical point of disease progression, and the incidence of further decompensation occurs up to more than 50%. Fuzheng Huayu tablet is used as a classic anti-hepatic fibrosis herbal compound. There is no randomized controlled trial (RCT) combining Fuzheng Huayu tablet with antiviral to prevent the occurrence of further decompensation. The aim of this study was to conduct a prospective randomized, double-blind, placebo-controlled, multicenter study to investigate its safety and efficacy in terms of the risk of further decompensation events in patients with first decompensation, and to fill the gap of evidence-based medicine in this field.

**Methods and analysis:**

This study aims to conduct a prospective, multicenter, double-blind, randomized, placebo-controlled trial. The planned sample size is 432 patients, with the ratio of the Fuzheng Huayu group to the placebo control group being 1:1. Participants will be randomly assigned to the two groups using a stratified block randomization method at the central level. The Fuzheng Huayu group (Fuzheng Huayu tablets combined with conventional antiviral therapy) and the placebo group (placebo combined with conventional antiviral therapy) will be treated for 48 weeks and followed up to 96 weeks. The study period will last for a total of 2 years. The cumulative incidence of further decompensation events within 1-year, the cumulative incidence of recompensation within 1-year, the incidence of liver failure, hepatocellular carcinoma, and liver disease-related deaths will be analyzed.

**Discussion:**

This study will be a prospective, randomized, double-blind, placebo-controlled, multicenter study to investigate the clinical benefit of Fuzheng Huayu tablets in the risk of further decompensation events in patients with first decompensation.

**Clinical Trial Registration:**

Clinical Trials.gov, study ID NCT07017426; International Traditional Medicine Clinical Trial Registry, registration number ITMCTR2025001365.

## 1 Introduction

Cirrhosis has a major impact on global health, increasing the risk of death by 5–10 times (Devarbhavi et al., 2023). Hepatitis B virus (HBV) infection is the leading cause of cirrhosis, and the 5-year survival rate of patients with further decompensated cirrhosis is less than 50%. Such patients often face a high risk of death due to complications such as portal hypertension, ascites, and hepatic encephalopathy, and the first decompensated event is a critical point for disease progression (Lee et al., 2021).

The Baveno VII Consensus Conference agreed on a definition of “further decompensation”. However, until now, the concept of further decompensation, with a higher mortality rate and how to reduce the incidence of further decompensation events, has not been supported by objective evidence (Reiberger and Hofer, 2023). A recent study has shown that the incidence of further decompensation in patients with cirrhosis with first decompensation in 5 years is as high as 51.5%, the risk of death reaches 15.6%, the incidence of further decompensation and death within 28 days is 7.3%, and the incidence of liver transplantation was 2.3% (D’Amico et al., 2024). Therefore, clear prevention of the risk of further decompensation in patients with first decompensation is a pressing clinical issue.

Although inhibition of viral replication by nucleoside (acid) analogs delays the progression of cirrhosis, antiviral therapy alone has limited reversal of established liver fibrosis and liver failure (Liaw et al., 2011). Studies have shown that even with a complete virologic response, patients with decompensated disease are still at high risk for continued progression of liver fibrosis and hepatocellular carcinoma, and there is an urgent need to explore combination therapeutic strategies to promote fibrosis reversal (Wang et al., 2022). At present, commercial Chinese polyherbal preparation (CCPP) such as Fuzheng Huayu, Biejia Ruangan, and Anluo Huaxian have exerted good curative effects in the field of anti-fibrosis, but the evidence level in the guidelines is not high (Yoshiji et al., 2021). Therefore, there is an urgent need to carry out high-level evidence-based evidence to provide evidence for traditional Chinese medicine (TCM) in the field of anti-fibrosis and anti-cirrhosis.

Fuzheng Huayu (FZHY) tablets is a classic Chinese medicine compound composed of Salvia miltiorrhiza Bunge [Lamiaceae; Salviae miltiorrhizae radix et rhizoma], Cordyceps sinensis (Berk.) Sacc [Ophiocordycipitaceae; Cordyceps], Schisandra chinensis (Turcz.) Baill [Schisandraceae; Schisandrae Chinensis Fructus], Gynostemma pentaphyllum (Thunb.) Makino [Cucurbitaceae; Gynostemmatis Herba], Pinus massoniana Lamb [Pinaceae; Pini Pollen], and Prunus persica (L.) Batsch [Rosaceae; Persicae Semen] (Table 1, Supplementary Materials 1–4) (Wang et al., 2018). Known as promoting blood circulation and removing blood stasis, nourishing the liver and replenishing essence. The preparation is clinically used to treat liver fibrosis and cirrhosis, and has multi-target mechanisms such as inhibiting the activation of hepatic stellate cells and improving the function of hepatic sinusoidal endothelium (Sun et al., 2022; Zha et al., 2024; Liu et al., 2009). Phase II in China showed that the reversal rate of liver fibrosis with FZHY tablets alone reached 52% (Liu et al., 2005). For patients with HBV-related cirrhosis, clinical retrospective analysis showed that the combination of FZHY tablets and antiviral drugs produced better efficacy and safety compared with antiviral monotherapy (Cheng et al., 2022; Li et al., 2021). At the same time, it was also found that the combination therapy of FZHY tablets and antiviral therapy in retrospective data can reduce the incidence of hepatocellular carcinoma (HCC) (Shi et al., 2023). Overall, FZHY tablets represent a promising comprehensive treatment option for the treatment of chronic liver disease, providing multiple benefits ranging from hepatitis and fibrosis to cirrhosis and HCC prevention. This is a prospective, randomized, double-blind, placebo-controlled, multicenter study to study the clinical evidence-based use of FZHY tablets in patients with first decompensation.

**TABLE 1:** Component of Fuzheng Huayu tablets.

Herbal name	Produced from	Doses (g)
Salvia miltiorrhiza bunge [lamiaceae]	Dry rhizoma	8
Cordyceps sinensis (berk.) sacc. [Ophiocordycipitaceae]	Dry composite	4
Schisandra chinensis (turcz.) baill. [Schisandraceae]	Dry fruit	2
Gynostemma pentaphyllum (thunb.) makino [cucurbitaceae]	Dry aboveground part	6
Pinus massoniana lamb. [Pinaceae]	Dry pollen	2
Prunus persica (L.) batsch [rosaceae]	Dry fruit	2

## 2 Methods and analysis

### 2.1 Investigational medications

Take Salvia miltiorrhiza Bunge, Prunus persica (L.) Batsch, and Gynostemma pentaphyllum (Thunb.) Makino, add 10 times and 8 times water respectively, decoct twice, the first time is 2 h, and the second time is 1.5 h, the decoction is filtered, the filtrate is combined, and concentrated to a relative density of 1.20 (50 °C–55 °C), let cool, add ethanol under stirring to make the alcohol content reach 70%, sit to precipitate, take the supernatant, filter, and concentrate the filtrate to a relative density of 1.1–1.2 (50 °C–55 °C), spare. In addition, take Cordyceps sinensis (Berk.) Sacc. and Schisandra chinensis (Turcz.) Baill., add 70% ethanol 10 times and 8 times respectively, heat and reflux twice, the first time is 1.5 h, and the second time is 1 h, merge the reflux solution, filter, and concentrate the filtrate to the relative density is 1.1–1.2 (50 °C–55 °C), set aside, then take Pinus massoniana Lamb. and add 50% ethanol 10 times and 8 times, soak twice at 50 °C, the first time is 4 h, the second time is 2 h, merge the leaching solution, concentrate to the relative density is 1.1–1.2 (50 °C–55 °C), merge with the above two spare concentrates, dry, add appropriate amount of auxiliary materials, mix well, granulate, press into a weight of 0.4 g per piece, and wrap in a film coating.

Fuzheng Huayu tablets is composed of six botanical drugs with functions of promoting blood circulation and removing blood stasis, replenishing essence and nourishing liver (Supplementary Material 8). Through modern pharmaceutical technology, the decoctions of Fuzheng Huayu tablets are then extracted, concentrated, dried, blended, granulated and pressed into tablets. Fuzheng Huayu tablets meet the national drug standard [YBZ19332005-2009Z] of the State Food and Drug Administration, and the approval number is [Sinopharm Zhunzi Z20050546].

### 2.2 Placebo control

Placebo preparation matched Fuzheng Huayu Tablets in appearance, color, smell, taste, weight and packaging. Herbal extracts or active pharmaceutical ingredients are not included. The same batch of excipients was used throughout the study to ensure consistency, and the inspection report is attached (Supplementary Material 9).

### 2.3 Trial design and setting

**FIGURE 1:**
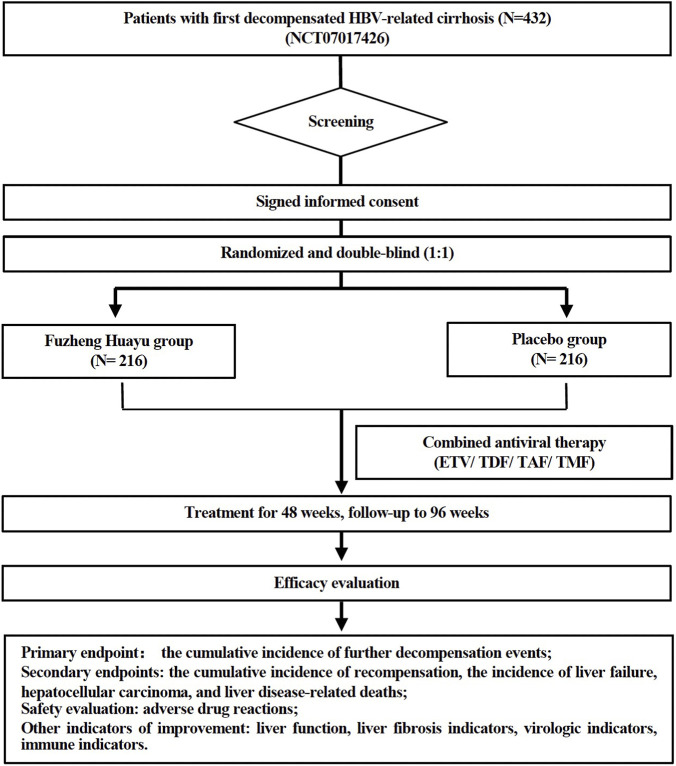
Flowchart of this study. ETV, entecavir; TDF, tenofovir disoproxil; TAF, propafenacortinofovir; TMF, emetinofovir.

This is a randomized, double-blind, placebo-controlled, multicenter trial designed to compare the incidence of recurrence of a first decompensation in patients treated with Fuzheng Huayu tablets [4 tablets three times a day] and placebo. Patients will be randomly assigned in a 1:1 ratio to receive either Fuzheng Huayu or placebo. Regular follow-up will be conducted every 12 weeks (Table 2). The flowchart of this trial is shown in Figure 1. Written informed consent was required for all participants before randomization (Supplementary Materials 5, 6). This protocol was prepared in accordance with the Standard Protocol Project, i.e., Recommendations for Intervention Trials (SPIRIT 2025) guidelines (Supplementary Material 7).

**TABLE 2:** The standard process of the form for the plan.

Study period	V1	V2	V3	V4	V5	V6	V7
	(Baseline)						
Study duration (Weeks)	0w	12w	24w	36w	48w	72w	96w
Informed consent Signing​​	●	​	​	​	​	​	​
Medical history taking	●	​	​	​	​	​	​
Physical examination and vital signs	●	●	●	●	●	●	●
Urinalysis	●	○	●	○	●	○	●
Complete blood count	●	●	●	●	●	●	●
Liver fibrosis	●	○	○	○	●	○	●
Liver function	●	●	●	●	●	●	●
Renal function	●	●	●	●	●	●	●
Quantitative hepatitis B surface antigen (HBsAg)	●	○	○	○	●	○	●
Quantitative hepatitis B e antigen (HBeAg)	●	○	○	○	●	○	●
HBV-DNA viral load	●	●	●	●	●	●	●
Coagulation function tests	●	●	●	●	●	●	●
Electrolyte panel	●	●	●	●	●	●	●
Thyroid function tests (TFTs)	●	○	○	○	●	○	●
Serum alpha-fetoprotein (AFP)​​	●	●	●	●	●	●	●
Abdominal ultrasound (US)​​	●	●	●	●	●	●	●
Liver stiffness Measurement (FibroScan)	●	○	●	○	●	●	●
Gastroscopy (for upper gastrointestinal Bleeding)​​	●	●	○	○	●	○	●
Liver biopsy Pathology​​	○	​	​	​	○	​	○
Immune-related biomarkers	●	​	●	​	●	​	●
Adverse events (AEs)	●	●	●	●	●	●	●

“●” indicates items that must be inspected/recorded, and “〇” indicates items that should be inspected/recorded as needed.

### 2.4 Participant selection

Participants who meet the following selection criteria will be included in the study: (1) voluntarily joining the group, able to understand and sign informed consent forms; (2) age range: 18–80 years old, gender not limited; (3) HBsAg positive for ≥6 months during screening; (4) complies with traditional Chinese medicine syndrome types: blood stasis obstructing collaterals, liver and kidney deficiency syndrome; (5) the first decompensated event, which meets the diagnostic criteria for decompensated cirrhosis in the “Diagnosis and Treatment Guidelines for Cirrhosis (2019 Edition)”: (a) having diagnostic evidence for cirrhosis; (b) complications related to portal hypertension may occur, such as ascites, esophageal and gastric variceal bleeding, hepatic encephalopathy, hepatorenal syndrome, etc.

We will exclude patients who meet any of the following criteria: (1) merge hepatitis A, C, D, E, and/or HIV infections; (2) merge autoimmune liver disease, alcoholic liver disease, drug-induced liver disease and other liver diseases; (3) patients with combined malignant tumors; (4) history of splenectomy and transjugular intrahepatic portosystemic shunt surgery; (5) individuals with neurological and psychiatric disorders, especially those with a history of depression, anxiety, mania, schizophrenia, or a family history of mental illness (especially those with a history of depression or tendencies towards depression); (6) individuals with severe heart, lung, kidney and other organ disorders; (7) individuals who plan to undergo organ transplantation or have already undergone organ transplantation; (8) pregnant or lactating women or those with fertility plans during the study period; (9) for those who are allergic to tonifying the body and removing blood stasis, nucleoside (acid) analogues, or drugs, or who meet any contraindications in the research drug instructions (10) individuals who have taken traditional Chinese medicine such as Fuzheng Huayu within the past 6 months (11) other situations that have participated in other intervention studies within the previous 3 months or are deemed unsuitable for inclusion by the researchers.

### 2.5 Recruitment and randomization

Prior to enrollment, all eligible participants will undergo an informed consent process. The researcher will provide comprehensive information about the purpose of the study, procedures, potential risks and benefits, and the voluntary nature of participation. Prior to providing written informed consent, participants will have the opportunity to ask questions and discuss participation with family members or counselors.

This multicenter study will use stratified randomization to generate a random sequence assigned to two groups. Stratified by center, within each center, participants meeting the inclusion and exclusion criteria will be assigned random numbers in the order of enrollment according to a pre-generated random coding table. A total of 432 drug numbers will be randomized in a 1:1 ratio to the Fuzheng Huayu group or the placebo-control group using stratified block randomization, with center as the stratification factor and a block size of 4. Prior to the start of the study, a statistician will generate the randomized coding table for participants (blinded randomization schedule) using SAS version 9.4. This schedule will be stamped with a special seal, and one copy will be kept at the research center. Another copy, the center code assignment (dispensing version), will be generated and given to the medication administrator. This dispensing version shows only the medication number and a group code (e.g., A/B); the correspondence between the group code and the actual treatment (Fuzheng Huayu or placebo) is concealed and known only to the statistician who holds the master randomization list. Based on this table, the medication administrator will assign medication numbers, and the assignment will be reviewed by another investigator. The administrator will then dispense the medication to the participant, record the date of assignment, and sign and file the form.

### 2.6 Intervention

Fuzheng Huayu group: Fuzheng Huayu 3 times a day, 4 tablets each time for 48 weeks. Conventional western medical treatment: symptomatic supportive therapy + antiviral therapy (recommended first-line potent low-resistance nucleoside (acid) analog therapy: entecavir (ETV)/tenofovir disoproxil (TDF)/propafenacort tenofovir (TAF)/emetinofovir (TMF).

Placebo group: in dosage form, shape, color, texture, odor, and dosage, exactly the same as the study drug, but without the active ingredient of the drug, 4 tablets 3 times a day for 48 weeks of treatment. Conventional western medical treatment: symptomatic supportive therapy + antiviral therapy (recommended first-line potent low-resistance nucleoside (acid) analog therapy: entecavir (ETV)/tenofovir disoproxil (TDF)/propafenacortinofovir (TAF)/emetinofovir (TMF).

### 2.7 Outcomes and measurements

Outcomes will be measured every 12 weeks the start of treatment to the end of follow-up is 96 weeks, meaning the total follow-up period is 96 weeks.

#### 2.7.1 Primary endpoint

The cumulative incidence of further decompensation events is defined as any of the following in cirrhotic patients 48 weeks after the first decompensation: (1) Development of a second portal hypertension - driven decompensating event (ascites, variceal bleeding, or hepatic encephalopathy) and/or jaundice; (2) Recurrent variceal bleeding, recurrent ascites (≥3 large-volume paracenteses within 1 year), recurrent encephalopathy, spontaneous bacterial peritonitis (SBP) and/or hepatorenal syndrome - acute kidney injury (HRS-AKI); (3) In patients with bleeding alone, development of ascites, encephalopathy, or jaundice after recovery from bleeding (not during the bleeding episode); (4) After the first decompensation, the new increase of total bilirubin >51.3 μmol/L (3 mg/dL) was judged as jaundice (de Franchis et al., 2022).

#### 2.7.2 Secondary endpoints

Secondary outcomes included rates of improvement in liver failure, hepatocellular carcinoma, liver disease-related mortality, liver fibrosis, cirrhosis, quality of life scores, improvement in laboratory markers, and adverse events.

#### 2.7.3 Safety assessments

The researchers closely observed the physical condition of the participants and checked them accordingly before enrolling in the group. The investigator will record all adverse events on the case report form (CRF) and report them to the person in charge. At the same time, any serious adverse events will be reported to the ethical committee of Beijing Ditan Hospital within 24 h, and the medical expert committee will determine whether the adverse events are related to FZHY. Participants have the right to withdraw from their consent to participate in the study at any time for any reason. The investigator will record the timing of the adverse event, its severity, the duration of the disease, and the appropriate actions taken in response to the adverse event.

### 2.8 Quality control and data management

Researchers will adhere to the clinical research system of quality control and quality assurance by adhering to their responsibility for the clinical research program, following standard operating procedures, and adhering to the clinical research system of quality control and quality assurance. Participants assignment in the study must conform to the designated random assignment scheme, and the processing grouping code for each participant will be stored confidentially by the statistical unit and the researcher.

All investigators will receive professional training, and investigators will follow good guideline clinical practices to ensure safety, blinding, and data quality. Investigators should ensure that all information is accurately and legally recorded in medical records and CRFs, and that two independent investigators assigned will double-check all CRFs. A synchronous electronic data acquisition (EDC) system will ensure real-time data entry and secure storage. The quality of data in the EDC system will be monitored by dedicated data management experts.

Ensure accurate recording of all laboratory metrics for data accuracy and authenticity. To assess data accuracy and procedural compliance, medical statisticians must accurately enter research data into reports and maintain all data management. Ethics committees at all research centers will oversee processes related to data access and analysis.

### 2.9 Withdrawal criteria

Participants will withdraw from the trial if: (1) the patient has experienced a serious adverse event. (2) Patients with severe changes or complications were associated with the disease during the trial. (3) Patients were unable to comply with the study plan, resulting in incomplete medical management records. (4) Patients who voluntarily requested to withdraw from the trial.

### 2.10 Sample size calculation

Sample size was calculated using a two-sided Z-test for two independent proportions with α = 0.05 and power = 80% (β = 0.20). Based on retrospective data, the 48-week cumulative incidence of further decompensation was 53.7% in patients who received commercial Chinese polyherbal preparation (CCPP) (including Fuzheng Huayu, Anluo Huaxian, or Fufang Biejia) plus antiviral therapy, and 67.5% in patients who received antiviral therapy alone. Applying these proportions to the current prospective trial (Fuzheng Huayu combined with antiviral therapy versus placebo combined with antiviral therapy) with a 1:1 allocation ratio, the required sample size per group was calculated as 194 using the formula. Accounting for a 10% dropout rate, we enrolled 216 patients per group, for a total of 432 patients. The calculation was performed using PASS 2021.

$$n=\frac{\left[{\left(Z_{\left\{\frac{\alpha }{2}\right\}}+Z_{\beta }\right)}^{2}\times \left(p1\left(1-p1\right)+p2\left(1-p2\right)\right)\right]}{{\left(p1-p2\right)}^{2}}$$

where $Z_{\left\{\frac{\alpha }{2}\right\}}$ = 1.96 (for α = 0.05), Z_β_ = 0.842 (for β = 0.20, power = 80%)

### 2.11 Data analysis plan

We used competitive risk analysis to ensure an accurate assessment of the cumulative incidence of specific events, Kaplan-Meier method was used for the survival curves of the two groups, and log-rank method was used for the comparison of the cumulative survival rates of the two groups. Continuous variables will be summarized as mean ± standard deviation or median with interquartile range. Within-group changes from baseline may be assessed using paired t-tests or Wilcoxon signed-rank tests, as appropriate. Between-group comparisons of changes from baseline will be performed using independent-sample t-tests or Wilcoxon rank-sum tests. The notational information of each visit of the two groups will be described statistically using frequency (percentage). Changes in the two groups before and after treatment were analyzed using the chi-square test. All statistical tests will be two-sided, and p < 0.05 will be considered statistically significant for the differences tested. Statistical analyses will be calculated using SPSS and SAS analysis software.

Analysis populations: Defined the ITT population as all randomized participants, analyzed according to their original randomized assignment regardless of whether they received the assigned treatment or completed the study. Defined the per-protocol (PP) population as participants who completed the 48-week treatment period without major protocol violations. In the ITT analysis, if the data related to the efficacy are missing, the method of last observation carried forward (LOCF) will be used to fill in.

Shedding analysis: Comparison of total shedding rate and shedding rate due to adverse events between the two groups will be done using chi-square test.

Balance analysis of baseline values: ANOVA or chi-square test will be used to compare demographic data and other baseline value indicators, and to measure how balanced the two groups are.

Adherence analysis: Adherence will be calculated as (actual dose taken/prescribed dose) × 100% over the 48-week treatment period. Good adherence is defined as adherence between 80% and 100% (inclusive). Participants with adherence <80% will be classified as having poor adherence. Participants with adherence >100% will be documented as protocol deviations and reviewed for safety; these participants will not be classified as good adherence and will be excluded from the per-protocol population. The chi-square test will be used to compare the proportion of participants with good adherence between the two groups.

Effectiveness analysis: For the primary time-to-event efficacy endpoint, a Cox proportional hazards model will be used to estimate the treatment effect, with treatment group as the main factor and study center included as a stratification factor or covariate, depending on the number of participants and events in each center. The Kaplan-Meier method and log-rank test will be used for descriptive and unadjusted comparisons. For binary efficacy outcomes, logistic regression adjusted for study center and relevant baseline covariates will be used. If some centers enroll only a small number of participants or have few outcome events, centers may be pooled according to prespecified rules before model adjustment.

Safety analysis: the chi-square test was used to compare the incidence of adverse events between the two groups and a list describing the adverse events that occurred in this study; the changes in normal/abnormal laboratory test results before and after the study as well as the relationship with the study drug in the event of abnormal changes.

### 2.12 Ethics and dissemination

This study is in accordance with the Declaration of Helsinki and has been approved by the ethical committee of Beijing Ditan Hospital (NO.DTEC-KY2025-071-02), and each individual provides informed consent. The results from this trial will be submitted for publication in peer-reviewed journals and will be presented at international conferences. The results of the study will make an important contribution to the optimization of therapeutic strategies for patients with cirrhosis.

## 3 Discussion

Patients with HBV-related cirrhosis enter a particularly vulnerable phase after the first decompensated event, with a high incidence of recurrent portal hypertension complications, liver failure and death. While potent nucleoside (acid) analogs achieve durable viral inhibition, their ability to reverse established fibrosis and prevent subsequent clinical deterioration after first decompensation remains limited. Therefore, this trial aimed to determine whether the combination of Fuzheng Huayu (FZHY) tablets in standard antiviral therapy reduces the risk of further decompensation as defined by Baveno VII, thereby providing a high level of evidence for clinical practice and potentially reshaping the therapeutic paradigm for decompensated cirrhosis.

Fuzheng Huayu (FZHY) tablets are widely employed in the clinical management of liver fibrosis and cirrhosis resulting from various chronic liver diseases. It has the effects of improving liver fibrosis-related indicators, reducing liver stiffness and portal hypertension, and reducing the incidence of hepatocellular carcinoma (HCC) in patients with liver cirrhosis and mortality (Zhou et al., 2024). Basic studies have shown that FZHY tablets can inhibit the activation of hepatic stellate cells, inhibit the inflammatory activation of macrophages, and promote liver regeneration (Ping et al., 2024; Ping et al., 2023; Chen et al., 2019). Clinically, the combination of FZHY tablets and antiviral drugs has been shown to produce synergistic effects. The study reported that the fibrosis reversal rate of FZHY tablets combined with entecavir in compensated cirrhosis was higher than that of entecavir monotherapy (54% vs. 39%) (Zhao et al., 2022). A multicenter retrospective cohort study showed a 12% reduction in 5-year cumulative incidence in HBV-HCC patients treated with FZHY tablets in combination with entecavir compared to patients treated with entecavir alone (9.8% vs. 21.8%) (Fan et al., 2024). These observations suggest that FZHY tablets not only improves fibrosis, but also delays disease progression by supplementing virological control achieved with antiviral therapy.

The present trial is designed to address important limitations of the existing evidence base and to enhance methodological rigor. A multicenter, double-blind, placebo-controlled randomized design minimized selection, performance, and outcome determination bias. Standard antiviral treatment in both groups enabled the assessment of the incremental benefit of FZHY tablets on the basis of optimal virological control. By focusing on the consensus-defined “further decompensation” reflecting key endpoints of irreversible disease progression, our study directly responds to the call for evidence-based strategies to prevent worsening after the first decompensation (D’Amico et al., 2024).

Several limitations should be taken into account when interpreting the design of this trial. First, the exclusion of cirrhotic patients with non-HBV such as alcohol-related or metabolic dysfunction-related fatty liver disease, may limit the generalization of the findings in the broader cirrhotic population. Second, the duration of follow-up, while sufficient to capture short-to medium-term decompensated events, may not fully reflect long-term outcomes, such as the development of HCC, which requires long-term observation after the trial period. Finally, placebo controls, while methodologically rigorous, raise ethical considerations in severe disease. However, the addition of optimal supportive treatment meets the criteria of the decompensated cirrhosis trial.

Despite these limitations, the key contribution of this study is to shift the evidence base for FZHY tablets from retrospective studies to the clinically decisive window, that is, after the first decompensation. This study is a registration of real-world evidence that will further verify the long-term safety and cost-effectiveness of traditional Chinese medicine (TCM). Further studies should also focus on optimizing the duration of treatment with FZHY tablets and identifying subgroups of patients most likely to benefit from treatment to identify patients most likely to benefit.

In conclusion, this study represents a critical step in integrating TCM into evidence-based cirrhosis management. FZHY tablets may become a safe and effective adjuvant to antiviral drugs for preventing further decompensation of HBV-related cirrhosis, addressing gaps in current guidelines. In addition to clinical benefits, our study also provides a paradigm for future “integrated Chinese and Western medicine” strategies for chronic liver diseases by showing how the multi-target approach of TCM complements Western therapies, contributing to precision medicine.
