# Supplementary material for: Fuzheng Huayu tablets reduces the risk of further decompensation after the first decompensation in patients with HBV-related cirrhosis: protocol for a randomized, double-blind, placebo-controlled, multicenter trial
Source: Front Pharmacol. 2026 Jul 2;17:1828944. doi: 10.3389/fphar.2026.1828944 (PMC13373875; doi:10.3389/fphar.2026.1828944)
Supplement: Supplementary file 2 [file Supplementaryfile9.pdf]

上海黄海制药有限责任公司
成品检验报告书

报告编号：HR2504009

品名	扶正化瘀空白片	产品代码	5443	规格	0.4g/片
批号	250402	数量	12,300盒		
生产日期	2025/03/28	包装规格	/		
有效期至	2028/03/27	报告日期	2025/04/17		
依据	扶正化瘀空白片(0.4g)质量标准SOP-RD-06-02-050				

检验项目	标准规定	检验结果
【性状】	本品为薄膜衣片，除去包衣后显棕色至棕褐色，味苦、涩	本品为薄膜衣片，除去包衣后显棕色至棕褐色，味苦、涩
【检查】		
重量差异	±5%	符合规定
崩解时限	＜60分钟	符合规定
【微生物限度】		
需氧菌总数	10^3cfu/g	<1×50cfu/g
霉菌和酵母菌数	10^2cfu/g	20cfu/g
大肠埃希菌	每1g不得检出	未检出

以下空白

结论：　本品按扶正化瘀空白片(0.4g)质量标准检验，结果符合规定

复核人：王[illegible]　　　　批准人：陈[illegible]

上海黄海制药有限责任公司 检验专用章

上海黄海制药有限责任公司
成品检验报告书

报告编号：HR2504008

品名	扶正化瘀空白片	产品代码	5443	规格	0.4g/片
批号	250401		数量	15,200盒	
生产日期	2025/03/28		包装规格	/	
有效期至	2028/03/27		报告日期	2025/04/17	
依据	扶正化瘀空白片(0.4g)质量标准SOP-RD-06-02-050				

检验项目	标准规定	检验结果
【性状】	本品为薄膜衣片，除去包衣后显棕色至棕褐色，味苦、涩	本品为薄膜衣片，除去包衣后显棕色至棕褐色，味苦、涩
【检查】		
重量差异	±5%	符合规定
崩解时限	＜60分钟	符合规定
【微生物限度】		
需氧菌总数	10^3cfu/g	<1×50cfu/g
霉菌和酵母菌数	10^2cfu/g	<1×10cfu/g
大肠埃希菌	每1g不得检出	未检出

以下空白

结论： 本品按扶正化瘀空白片(0.4g)质量标准检验，结果符合规定

复核人：王慧　　　　批准人：陈鑫
