# Supplementary material for: Fuzheng Huayu tablets reduces the risk of further decompensation after the first decompensation in patients with HBV-related cirrhosis: protocol for a randomized, double-blind, placebo-controlled, multicenter trial
Source: Front Pharmacol. 2026 Jul 2;17:1828944. doi: 10.3389/fphar.2026.1828944 (PMC13373875; doi:10.3389/fphar.2026.1828944)
Supplement: Supplementary file 3 [file Supplementaryfile8.pdf]

上海黄海制药有限责任公司
成品检验报告书

报告编号：HF2504102

品名	扶正化瘀片	产品代码	5403	规格	0.4g
批号	250334		数量	18,000盒	
生产日期	2025/03/15		包装规格	48片/瓶×10盒/条×10条/箱	
有效期至	2028/03/14		报告日期	2025/04/09	
依据	YBZ19332005-2009Z国家食品药品监督管理局国家药品标准				

检验项目	标准规定	检验结果
【性状】	本品为薄膜衣片，除去包衣后显棕色至棕褐色，味苦、涩	本品为薄膜衣片，除去包衣后显棕色至棕褐色，味苦、涩
【鉴别】		
薄层鉴别（1）	应与腺苷对照品在相应的位置显相同颜色的斑点	与腺苷对照品在相应的位置显相同颜色的斑点
薄层鉴别（2）	应与原儿茶醛对照品在相应的位置显相同颜色的斑点	与原儿茶醛对照品在相应的位置显相同颜色的斑点
薄层鉴别（3）	应与绞股蓝皂苷对照品在相应的位置显相同颜色的斑点	与绞股蓝皂苷对照品在相应的位置显相同颜色的斑点
【检查】		
重量差异	±5%	符合规定
崩解时限	＜60分钟	符合规定
【微生物限度】		
需氧菌总数	10^3cfu/g	50cfu/g
霉菌和酵母菌数	10^2cfu/g	<1×10cfu/g
大肠埃希菌	每1g不得检出	未检出
【含量测定】		
丹参素钠	≥1.10mg/片	2.76mg/片
腺　苷	≥0.40mg/片	0.75mg/片

以下空白

结论：　本品按扶正化瘀片(0.4g)质量标准检验，结果符合规定

上海黄海制药有限责任公司 检验专用章

复核人：王慧

批准人：陈鑫

上海黄海制药有限责任公司
成品检验报告书

报告编号：HR2505013

品名	扶正化瘀片	产品代码	5403	规格	0.4g
批号	250335(科研用)		数量	9,500盒	
生产日期	2025/03/15		包装规格	48片/瓶×10盒/条×10条/箱	
有效期至	2028/03/14		报告日期	2025/05/19	
依据	YBZ19332005-2009Z国家食品药品监督管理局国家药品标准				

检验项目	标准规定	检验结果
【性状】	本品为薄膜衣片，除去包衣后显棕色至棕褐色，味苦、涩	本品为薄膜衣片，除去包衣后显棕色至棕褐色，味苦、涩
【鉴别】		
薄层鉴别（1）	应与腺苷对照品在相应的位置显相同颜色的斑点	与腺苷对照品在相应的位置显相同颜色的斑点
薄层鉴别（2）	应与原儿茶醛对照品在相应的位置显相同颜色的斑点	与原儿茶醛对照品在相应的位置显相同颜色的斑点
薄层鉴别（3）	应与绞股蓝皂苷对照品在相应的位置显相同颜色的斑点	与绞股蓝皂苷对照品在相应的位置显相同颜色的斑点
【检查】		
重量差异	±5%	符合规定
崩解时限	＜60分钟	符合规定
【微生物限度】		
需氧菌总数	10^3cfu/g	<1×50cfu/g
霉菌和酵母菌数	10^2cfu/g	<1×10cfu/g
大肠埃希菌	每1g不得检出	未检出
【含量测定】		
丹参素钠	≥1.10mg/片	2.86mg/片
腺　苷	≥0.40mg/片	0.86mg/片

以下空白

结论：　本品按扶正化瘀片(0.4g)质量标准检验，结果符合规定

上海黄海制药有限责任公司 检验专用章

复核人：王琵　　　　　　　　批准人：陈鑫
