# Supplementary material for: Fuzheng Huayu tablets reduces the risk of further decompensation after the first decompensation in patients with HBV-related cirrhosis: protocol for a randomized, double-blind, placebo-controlled, multicenter trial
Source: Front Pharmacol. 2026 Jul 2;17:1828944. doi: 10.3389/fphar.2026.1828944 (PMC13373875; doi:10.3389/fphar.2026.1828944)
Supplement: Supplementary file 4 [file Supplementaryfile1.doc]

**1. The composition of Fuzheng Huayu Tablets.**


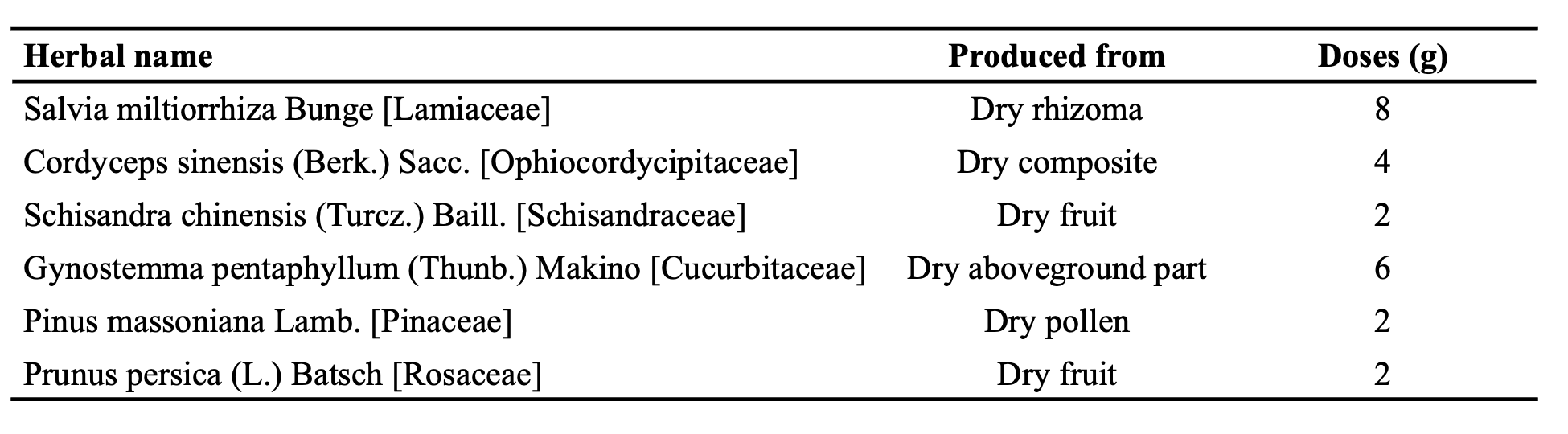


**2、The preparation of Fuzheng Huayu Tablets.**

A total of 1998g of the raw herb material for Fuzheng Huayu Tablets, first take Salvia miltiorrhiza Bunge, Prunus persica (L.) Batsch, and Gynostemma pentaphyllum (Thunb.) Makino, add 10 times and 8 times of water respectively, decoct twice, the first time is 2 h, and the second time is 1.5 h, the decoction is filtered, the filtrate is combined, concentrated to a relative density of 1.20 (50 °C to 55 °C), let it cool, add ethanol under stirring to make the alcohol content reach 70%, sit and settle, take the supernatant, filter, and concentrate the filtrate to a relative density of 1.1 ~ 1.2 (50 °C to 55 °C), set aside. In addition, take Cordyceps sinensis (Berk.) Sacc. and Schisandra chinensis (Turcz.) Baill., add 70% ethanol 10 times and 8 times respectively, heat and reflux twice, the first time is 1.5 h, and the second time is 1 h, merge the reflux solution, filter, and concentrate the filtrate to the relative density is 1.1-1.2 (50 °C-55 °C), set aside. Then take Pinus massoniana Lamb. and add 50% ethanol 10 times and 8 times, soak twice at 50 °C, the first time is 4 h, the second time is 2 h, merge the leaching solution, concentrate to the relative density is 1.1-1.2 (50 °C-55 °C), merge with the above two spare concentrates, dry, add an appropriate amount of auxiliary materials, mix well, granulate, press into 1000 pieces (weight 0.4 g per piece), and wrap a film coating, namely.
