# Supplementary material for: Fuzheng Huayu tablets reduces the risk of further decompensation after the first decompensation in patients with HBV-related cirrhosis: protocol for a randomized, double-blind, placebo-controlled, multicenter trial
Source: Front Pharmacol. 2026 Jul 2;17:1828944. doi: 10.3389/fphar.2026.1828944 (PMC13373875; doi:10.3389/fphar.2026.1828944)
Supplement: Supplementary file 5 [file Supplementaryfile2.doc]

**Analysis of Fuzheng Huayu Tablets by UHPLC-MS/MS**

**Materials and methods**

**1. Sample processing**

A sample of 200mg was taken and placed in a 15mL centrifuge tube, 10 ml of 50% methanol water solution (v: v, water: methanol = 50: 50) was added, ultrasound for 30min, supernatant of 1mL was taken and placed in a centrifuge tube, centrifuged at 14000rpm for 5min, supernatant was taken through a 0.22 um microporous filter membrane, placed in a sample bottle, and analyzed by UHPLC-MS/MS. Blank samples were treated under the same conditions.

**2. Liquid phase condition**

An ACQUITY UPLC HSS T3 column (2.1 × 100 mm, 1.8 μm) was used with a column temperature of 35 °C, an injection volume of 10 μL, and a flow rate of 0.3 mL/min. The mobile phases were: phase A (deionized water with 0.1% formic acid) and phase B (acetonitrile with 0.1% formic acid), using a gradient elution procedure: 0 min, 100% A; 10 min, 70% A; 25 min, 60% A; 30 min, 50% A; 40 min, 30% A; 45 min, 0% A; 60-60.5 min, linearly changing from 0% A to 100% A, and ending the run at 70 min.

**3. Mass spectrum condition**

Mass spectrometry data acquisition was performed using Q Exactive Orbitrap high-resolution mass spectrometry, detection mode was Full MS-ddMS2, positive and negative ion modes were scanned separately, scanning range was 100-1200, MS1 resolution was set to 70000, MS2 resolution was set to 17500, ion source voltage was 3.2 kV, capillary ion transport tube temperature was 320 °C, auxiliary gas heating temperature was 350 °C, Sheath gas flow rate was 40 L/min, auxiliary gas flow rate was 15 L/min, AGC Target was set to 1e6, TopN was set to 5, and the collision energy triggering MS2 scanning was set to 30, 40, 50 using stepped fragmentation voltage NCE.

**Results**

**1.The TIC scans in positive and negative ion modes are shown in Figure 1 and 2, respectively.**


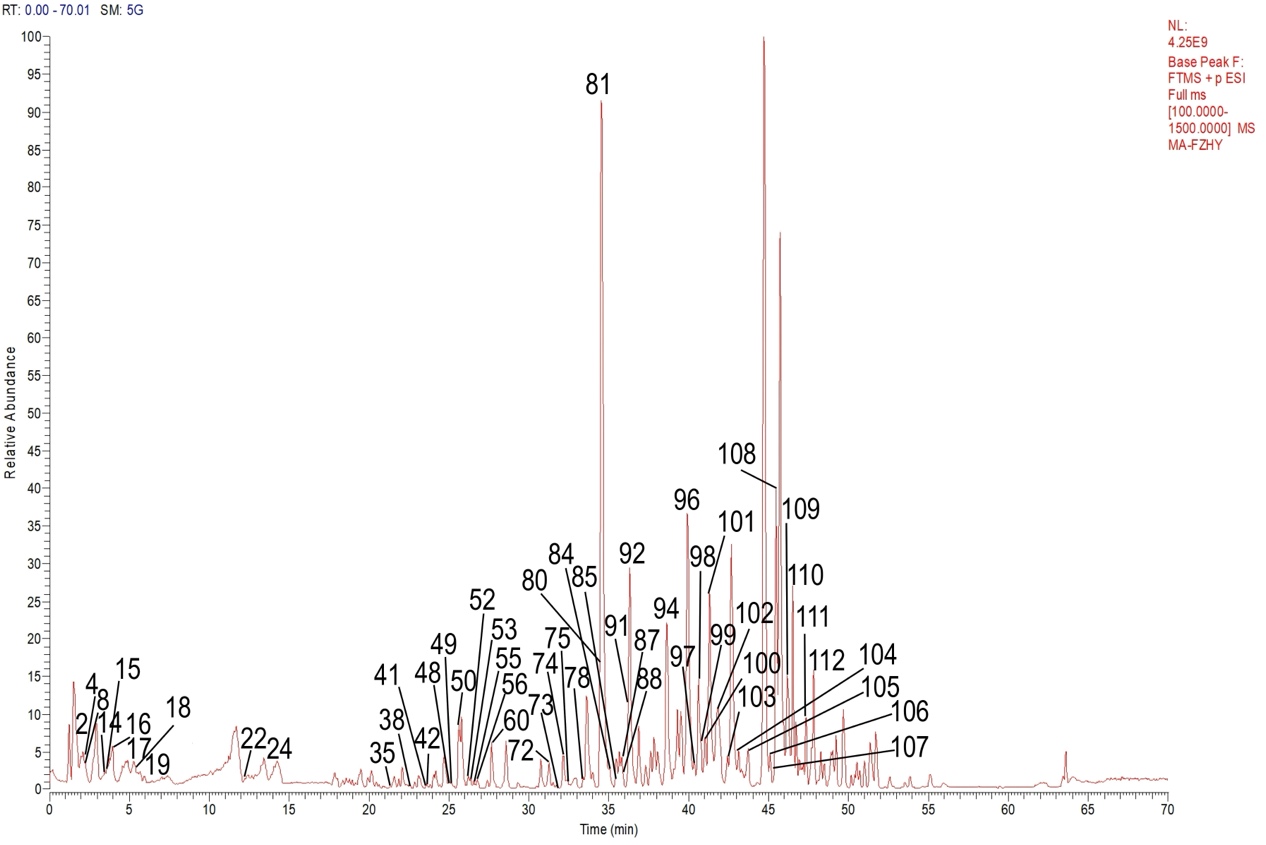


Figure 1 TIC diagram of Fuzheng Huayu sample in positive ion mode


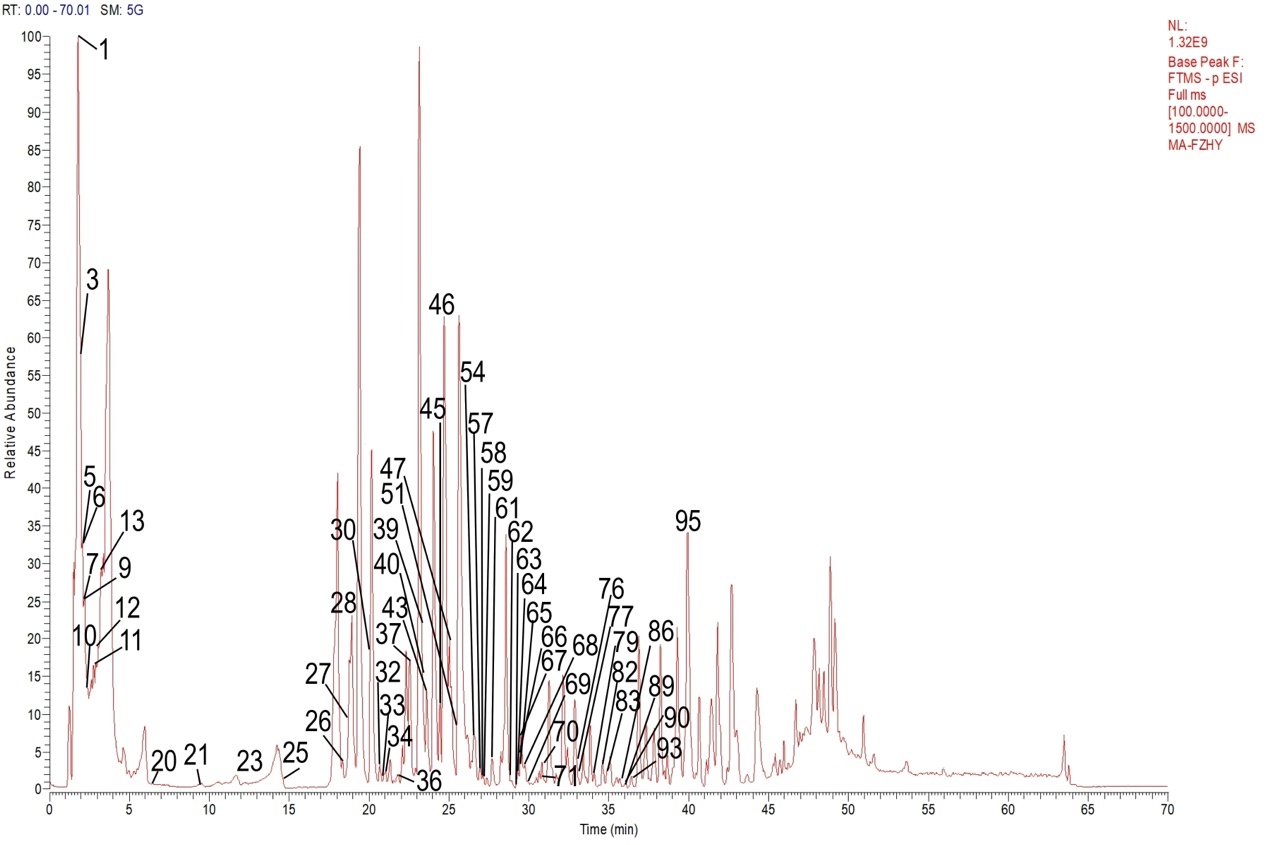


Figure 2 TIC diagram of Fuzheng Huayu sample in negative ion mode

**2. Compound identification results**

Compound Discoverer 3.2 software was used to process the original mass spectrometry data and extract the characteristic peaks. In the process of compound identification, the mass deviation thresholds for element composition matching, molecular formula prediction and isotope distribution matching of characteristic peaks are all set at 5 ppm.

Subsequently, the extracted characteristic peaks were identified by comprehensively using the mzCloud online database, the local self-built mzVault traditional Chinese medicine natural product database, and the comparison standard products. The screening criteria for positive identification results were set as mass deviation < 5 ppm, consistent with the characteristic isotope distribution pattern, retention time deviation within 0.2 min from the control standard, consistent with mass spectrum fragment peaks, and the best match score in mzVault database > 70 points.

After the above-mentioned process identification, combined with manual verification and elimination of repeated results, 112 chemical constituents were identified from the roots of torch flowers in this study. Among them, 42 components were confirmed by comparison of retention time and mass spectrogram with the corresponding standard products. The specific identification results are shown in Table 1.

**Table 1 Sample identification compound information**

| **Number** | **RT/min** | **Ion model** | **Measured mass/Da** | **Calculated mass/Da** | **Error/ppm** | **Formula** | **Name** | **Peak Area** |
| --- | --- | --- | --- | --- | --- | --- | --- | --- |
| 1 | 1.962 | [M-H]⁻ | 181.07166 | 182.07894 | -0.53 | C₆H₁₄O₆ | Mannitol | 1633423210 |
| 2 | 2.02 | [M+H]⁺ | 118.0864 | 117.07912 | 1.24 | C₅H₁₁NO₂ | L-Valine | 2338259531 |
| 3 | 2.057 | [M-H]⁻ | 191.05603 | 192.06333 | -0.3 | C₇H₁₂O₆ | Quinic acid | 532224214.8 |
| 4 | 2.067 | [M+H]⁺ | 162.11266 | 161.10538 | 1.16 | C₇H₁₅NO₃ | L(-)-Carnitine | 242591765.1 |
| 5 | 2.071 | [M-H]⁻ | 549.16736 | 550.17455 | -0.68 | C₁₈H₃₂O₁₆ | Raffinose | 201571436.6 |
| 6 | 2.083 | [M-H]⁻ | 341.10866 | 342.11596 | -0.74 | C₁₂H₂₂O₁₁ | Sucrose | 539638265.1 |
| 7 | 2.09 | [2M+FA-H]⁻ | 701.19142 | 328.09973 | 0.37 | C₁₂H₂₂O₁₁ | Lactose | 85569472.76 |
| 8 | 2.093 | [M+H]⁺ | 116.07079 | 115.06352 | 1.63 | C₅H₉NO₂ | 2-Pyrrolidinecarboxylic acid | 7201442427 |
| 9 | 2.099 | [M-H]⁻ | 115.00347 | 116.01075 | -1.77 | C₄H₄O₄ | Maleic acid* | 380519841.4 |
| 10 | 2.26 | [M-H]⁻ | 191.01962 | 192.0269 | -0.53 | C₆H₈O₇ | Citric acid | 27663178610 |
| 11 | 2.905 | [M-H]⁻ | 173.04532 | 174.0526 | -1.27 | C₇H₁₀O₅ | Shikimic acid* | 540964408.9 |
| 12 | 2.966 | [M+FA-H]⁻ | 711.22017 | 666.2219 | 0.06 | C₂₄H₄₂O₂₁ | Stachyose | 913627523 |
| 13 | 3.083 | [M+FA-H]⁻ | 549.16736 | 504.1691 | 0.14 | C₁₈H₃₂O₁₆ | Manninotriose | 256901979.5 |
| 14 | 3.458 | [M+H]⁺ | 124.0395 | 123.03222 | 1.59 | C₆H₅NO₂ | Nicotinic acid | 1659263638 |
| 15 | 3.498 | [M+H]⁺ | 112.05068 | 111.0434 | 1.28 | C₄H₅N₃O | Cytosine | 244068100.9 |
| 16 | 3.95 | [M+H]⁺ | 123.05541 | 122.04813 | 0.98 | C₆H₆N₂O | Nicotinamide | 334498260.2 |
| 17 | 5.182 | [M+H]⁺ | 182.08139 | 181.07411 | 1.2 | C₉H₁₁NO₃ | L-Tyrosine | 1113869405 |
| 18 | 5.287 | [M+H]⁺ | 132.10207 | 131.09479 | 1.23 | C₆H₁₃NO₂ | L-Leucine | 5995873851 |
| 19 | 6.127 | [M+NH₄]⁺ | 182.08138 | 164.04755 | 1.23 | C₉H₈O₃ | p-Coumaric acid* | 2278235565 |
| 20 | 6.399 | [M-H]⁻ | 243.06206 | 244.06939 | -0.62 | C₉H₁₂N₂O₆ | Uridine | 1920115398 |
| 21 | 8.618 | [M-H]⁻ | 169.01407 | 170.02136 | -0.98 | C₇H₆O₅ | Gallic acid* | 83829621.47 |
| 22 | 12.107 | [M+H]⁺ | 268.10424 | 267.09695 | 0.72 | C₁₀H₁₃N₅O₄ | Adenosine | 12524530438 |
| 23 | 12.115 | [M-H]⁻ | 134.0471 | 135.05463 | 1.02 | C₅H₅N₅ | Adenine | 670360252.6 |
| 24 | 14.727 | [M+H]⁺ | 152.0569 | 151.04961 | 1.31 | C₅H₅N₅O | Guanine | 4824042946 |
| 25 | 14.727 | [M-H]⁻ | 282.08428 | 283.09171 | 0.15 | C₁₀H₁₃N₅O₅ | Guanosine | 3817173887 |
| 26 | 18.352 | [M-H]⁻ | 153.01917 | 154.02645 | -1.04 | C₇H₆O₄ | Protocatechuic acid* | 556181497 |
| 27 | 18.493 | [M-H]⁻ | 197.04536 | 198.05268 | -0.73 | C₉H₁₀O₅ | Danshensu* | 5980432057 |
| 28 | 18.892 | [M-H]⁻ | 305.06647 | 306.07391 | -0.14 | C₁₅H₁₄O₇ | Epigallocatechin* | 99101395.89 |
| 29 | 19.861 | [M-H]⁻ | 137.02421 | 138.03153 | -1.18 | C₇H₆O₃ | Protocatechualdehyde* | 14488067838 |
| 30 | 19.98 | [M-H+TFA]⁻ | 355.10324 | 242.11735 | -0.42 | C₁₆H₂₀O₉ | Gentiopicrin* | 3775348.33 |
| 31 | 20.165 | [M-H]⁻ | 153.01917 | 154.02645 | -1.06 | C₇H₆O₄ | Gentisic acid* | 52548986.97 |
| 32 | 20.627 | [M+FA-H]⁻ | 502.15659 | 457.15842 | 0.01 | C₂₀H₂₇NO₁₁ | Amygdalin* | 6915202350 |
| 33 | 20.631 | [M-H]⁻ | 193.05038 | 194.05771 | -1.04 | C₁₀H₁₀O₄ | Ferulic acid* | 203460366.5 |
| 34 | 21.07 | [M-H]⁻ | 177.01926 | 178.02661 | 0.02 | C₉H₆O₄ | Esculetin | 62847109.04 |
| 35 | 21.111 | [M+H]⁺ | 197.08107 | 196.07378 | 1.15 | C₁₀H₁₂O₄ | Homoveratrumic acid | 8367255.477 |
| 36 | 21.619 | [M-H]⁻ | 121.02935 | 122.03666 | -1 | C₇H₆O₂ | p-Hydroxybenzaldehyde | 153959534 |
| 37 | 22.546 | [M-H]⁻ | 609.14606 | 610.15342 | 0.06 | C₂₇H₃₀O₁₆ | Rutin* | 683704951.8 |
| 38 | 22.547 | [M+H]⁺ | 465.103 | 464.09572 | 0.53 | C₂₁H₂₀O₁₂ | Isoquercitrin* | 57527522.25 |
| 39 | 23.317 | [M-H]⁻ | 593.15161 | 594.15899 | 0.88 | C₂₇H₃₀O₁₅ | Kaempferol-3-O-rutinoside* | 29811169.76 |
| 40 | 23.475 | [M-H]⁻ | 623.16205 | 624.16946 | 0.68 | C₂₈H₃₂O₁₆ | Narcissoside* | 40925884.31 |
| 41 | 23.602 | [M+H]⁺ | 147.04408 | 146.03681 | 0.18 | C₉H₆O₂ | Coumarin* | 95521802.96 |
| 42 | 23.612 | [M+H]⁺ | 193.04964 | 192.04236 | 0.51 | C₁₀H₈O₄ | 4-Methyl-6,7-dihydroxycoumarin | 108344641.9 |
| 43 | 23.8 | [M-H]⁻ | 447.09344 | 448.10072 | 0.36 | C₂₁H₂₀O₁₁ | Astragalin* | 30430781.08 |
| 44 | 24.496 | [2M-H]⁻ | 359.07715 | 180.04221 | -0.25 | C₉H₈O₄ | Caffeic acid* | 5821894019 |
| 45 | 24.576 | [M-H]⁻ | 187.09742 | 188.1047 | -0.87 | C₉H₁₆O₄ | Azelaic acid | 292657731.6 |
| 46 | 24.885 | [M+FA-H]⁻ | 991.54901 | 946.55075 | 0.67 | C₄₈H₈₂O₁₈ | Ginsenoside Re* | 40422051.82 |
| 47 | 24.924 | [M-H]⁻ | 845.49072 | 846.498 | 0.88 | C₄₂H₇₂O₁₄ | Ginsenoside Rg1* | 28991880.4 |
| 48 | 25.166 | [M+H]⁺ | 539.11847 | 538.11119 | 0.12 | C₂₇H₂₂O₁₂ | Lithospermic acid* | 775243037.8 |
| 49 | 25.247 | [M+H]⁺ | 447.09192 | 446.08466 | -0.57 | C₂₁H₁₈O₁₁ | Baicalin* | 260945476.8 |
| 50 | 25.653 | [M+H]⁺ | 191.10676 | 190.09949 | 0.6 | C₁₂H₁₄O₂ | Ligustilide* | 6836620.866 |
| 51 | 26.106 | [M-H]⁻ | 493.1138 | 494.12104 | -0.51 | C₂₆H₂₂O₁₀ | Salvianolic acid A* | 16372071541 |
| 52 | 26.204 | [M+H]⁺ | 175.03902 | 174.03151 | -1.03 | C₁₀H₆O₃ | Lawsone | 41866594.37 |
| 53 | 26.251 | [M+H]⁺ | 255.06513 | 254.05783 | -0.3 | C₁₅H₁₀O₄ | Daidzein* | 3986628617 |
| 54 | 26.44 | [M-H]⁻ | 373.09277 | 374.10005 | -0.31 | C₁₉H₁₈O₈ | Methyl rosmarinate | 98599353.86 |
| 55 | 26.524 | [M+H]⁺ | 247.13299 | 246.12572 | 0.51 | C₁₅H₁₈O₃ | Arglabin | 14488605.95 |
| 56 | 26.67 | [M+H]⁺ | 285.07593 | 284.06862 | 0.52 | C₁₆H₁₂O₅ | Emodin-3-methyl ether/Physcion* | 714752254.2 |
| 57 | 27.048 | [M-H]⁻ | 943.49036 | 944.49763 | 0.67 | C₄₆H₇₄O₁₇ | Gypenoside A* | 24684925.84 |
| 58 | 27.096 | [M-H]⁻ | 491.09848 | 492.10575 | 0.21 | C₂₆H₂₀O₁₀ | Salvianolic acid C* | 1046600343 |
| 59 | 27.097 | [M-H]⁻ | 301.03529 | 302.04264 | -0.06 | C₁₅H₁₀O₇ | Quercetin* | 434072320.1 |
| 60 | 27.518 | [M+H]⁺ | 493.13446 | 492.12703 | 0.51 | C₂₃H₂₄O₁₂ | Aurantio-obtusin β-D-glucoside | 42662956.04 |
| 61 | 27.751 | [M-H]⁻ | 207.06621 | 208.07354 | -0.08 | C₁₁H₁₂O₄ | Ethyl caffeate | 33784694.02 |
| 62 | 28.891 | [M-H]⁻ | 271.06113 | 272.06846 | -0.05 | C₁₅H₁₂O₅ | Naringenin chalcone | 854601480.1 |
| 63 | 29.025 | [M-H]⁻ | 269.04544 | 270.05272 | -0.39 | C₁₅H₁₀O₅ | Genistein | 4179897862 |
| 64 | 29.17 | [M-2H]²⁻ | 1045.55847 | 2093.1315 | 0.67 | C₅₂H₈₆O₂₁ | Gypenoside XLIX | 32623499.66 |
| 65 | 29.357 | [M-H]⁻ | 269.08194 | 270.08924 | 0.11 | C₁₆H₁₄O₄ | Medicarpin | 124876017.1 |
| 66 | 29.405 | [M-H]⁻ | 285.04044 | 286.04775 | 0.05 | C₁₅H₁₀O₆ | Kaempferol* | 134321441.3 |
| 67 | 29.522 | [M+FA-H]⁻ | 829.49609 | 784.49763 | 0.43 | C₄₂H₇₂O₁₃ | Ginsenoside Rg2* | 42586115.93 |
| 68 | 29.799 | [M-H]⁻ | 315.05106 | 316.05834 | 0.13 | C₁₆H₁₂O₇ | Isorhamnetin* | 81293459.74 |
| 69 | 29.939 | [M-H]⁻ | 1123.59021 | 1124.59745 | 0.88 | C₅₃H₉₀O₂₂ | Ginsenoside Rc | 23542852.9 |
| 70 | 30.98 | [M-H]⁻ | 683.43762 | 684.4449 | -0.13 | C₃₆H₆₂O₉ | Ginsenoside F1 | 46832756.02 |
| 71 | 30.982 | [M+FA-H]⁻ | 991.54822 | 946.55004 | -0.08 | C₄₈H₈₂O₁₈ | Gypenoside XVII | 56465034.02 |
| 72 | 31.209 | [M+H]⁺ | 151.11186 | 150.10457 | 0.68 | C₁₀H₁₄O | 2-Adamantanone | 1075966778 |
| 73 | 31.935 | [M+H]⁺ | 251.20062 | 250.19335 | 0.26 | C₁₆H₂₆O₂ | Clareolide | 46640488.5 |
| 74 | 32.154 | [M+H]⁺ | 247.09666 | 246.08948 | 1.09 | C₁₄H₁₄O₄ | Decursinol | 130153483.2 |
| 75 | 32.248 | [M+H]⁺ | 231.13814 | 230.13085 | 0.75 | C₁₅H₁₈O₂ | Atractylenolide I* | 74365421.64 |
| 76 | 33.087 | [M-H]⁻ | 961.53729 | 962.54457 | -0.31 | C₄₇H₈₀O₁₇ | Notoginsenoside Fe | 50946403.16 |
| 77 | 33.131 | [M-H]⁻ | 913.47919 | 914.48646 | 0.88 | C₄₅H₇₂O₁₆ | Dioscin* | 5678789.695 |
| 78 | 33.334 | [M+H-H₂O]⁺ | 423.36221 | 440.36553 | 0.22 | C₃₀H₄₈O₂ | Roburic acid | 629948265.6 |
| 79 | 34.169 | [M-H]⁻ | 373.09277 | 374.10005 | -0.31 | C₁₉H₁₈O₈ | Casticin | 7756154.702 |
| 80 | 34.422 | [M+H]⁺ | 245.11746 | 244.11019 | 0.99 | C₁₅H₁₆O₃ | Linderalactone | 117201826.6 |
| 81 | 34.591 | [M+H]⁺ | 327.1593 | 326.15284 | 3.17 | C₂₀H₂₂O₄ | Dehydrodiisoeugenol | 36222729.17 |
| 82 | 34.685 | [M-H]⁻ | 829.49554 | 830.50282 | -0.42 | C₄₂H₇₂O₁₃ | Ginsenoside F2 | 47606933.62 |
| 83 | 34.787 | [M+FA-H]⁻ | 845.49054 | 800.49224 | 0.04 | C₄₂H₇₂O₁₄ | Ginsenoside Rf | 18521237.25 |
| 84 | 35.011 | [M+H-H₂O]⁺ | 415.21161 | 432.21486 | 0.13 | C₂₄H₃₂O₇ | Schisandrin | 48856067275 |
| 85 | 35.216 | [M+H]⁺ | 331.08133 | 330.07404 | 0.28 | C₁₇H₁₄O₇ | Jaceosidin | 692009376.3 |
| 86 | 35.841 | [M-H]⁻ | 633.40088 | 634.40816 | 0.11 | C₃₆H₅₈O₉ | Ecliptasaponin A | 117428309.7 |
| 87 | 35.935 | [M+NH₄]⁺ | 548.24951 | 530.21569 | 0.94 | C₂₈H₃₄O₁₀ | Gomisin D | 1787455816 |
| 88 | 35.965 | [M+H]⁺ | 469.33163 | 468.32432 | 0.78 | C₃₀H₄₄O₄ | Glabrolide | 39982219.99 |
| 89 | 36.076 | [M-H]⁻ | 701.28168 | 702.28895 | 0.28 | C₃₆H₄₆O₁₄ | Taccalonolide A | 15442130.41 |
| 90 | 36.215 | [M+FA-H]⁻ | 829.49554 | 784.49727 | -0.03 | C₄₂H₇₂O₁₃ | 20(R)-Ginsenoside Rg3 | 73848803.94 |
| 91 | 36.217 | [M+H-H₂O]⁺ | 407.36752 | 424.37089 | 0.87 | C₃₀H₄₈O | Lupenone | 43302404.5 |
| 92 | 36.279 | [M+H+MeOH]⁺ | 389.19617 | 356.16267 | 0.72 | C₂₂H₂₈O₆ | Gomisin J | 1847358853 |
| 93 | 36.527 | [M+FA-H]⁻ | 829.49554 | 784.49727 | -0.03 | C₄₂H₇₂O₁₃ | Ginsenoside Rg3* | 59193617.64 |
| 94 | 38.528 | [M+H]⁺ | 279.10184 | 278.09456 | 0.94 | C₁₈H₁₄O₃ | Dihydrotanshinone I | 2435448698 |
| 95 | 39.798 | [M-H]⁻ | 315.25366 | 316.26094 | 1.2 | C₁₇H₃₄O₂ | Methyl hexadecanoate | 517599556.1 |
| 96 | 39.807 | [M+H]⁺ | 151.11186 | 150.10457 | 0.7 | C₁₀H₁₄O | Perillene | 103674458.6 |
| 97 | 40.279 | [M+H]⁺ | 554.23901 | 553.23174 | -1.77 | C₃₀H₃₂O₉ | Gomisin G | 777112133 |
| 98 | 40.689 | [M+H]⁺ | 219.17438 | 218.1671 | 0.18 | C₁₅H₂₂O | (+)-Nootkatone | 173273839.1 |
| 99 | 40.799 | [M+H]⁺ | 315.1958 | 314.18849 | 0.92 | C₂₀H₂₆O₃ | Kahweol | 1461142023 |
| 100 | 41.087 | [M+H]⁺ | 403.21188 | 402.2046 | 0.9 | C₂₃H₃₀O₆ | Schisanhenol | 6779861291 |
| 101 | 41.493 | [M+H]⁺ | 554.23901 | 553.23174 | 0.75 | C₃₀H₃₂O₉ | Schisantherin A* | 2393154968 |
| 102 | 41.762 | [M+NH₄]⁺ | 532.25452 | 514.22067 | 0.75 | C₂₈H₃₄O₉ | Schizandrol B* | 5661051973 |
| 103 | 42.188 | [M+H]⁺ | 297.14868 | 296.1414 | 0.52 | C₁₉H₂₀O₃ | Cryptotanshinone* | 4161334459 |
| 104 | 42.944 | [M+H]⁺ | 219.17436 | 218.16709 | 0.09 | C₁₅H₂₂O | α-Cyperone | 154658923.9 |
| 105 | 43.498 | [M+H]⁺ | 303.23187 | 302.2246 | 0.05 | C₂₀H₃₀O₂ | Abietic Acid | 175301013.4 |
| 106 | 45.129 | [M+H]⁺ | 219.17436 | 218.16712 | 0.24 | C₁₅H₂₂O | Germacrone | 1238034028 |
| 107 | 45.192 | [M+H]⁺ | 417.22726 | 416.21998 | 0.21 | C₂₄H₃₂O₆ | Schizandrin A* | 44240378091 |
| 108 | 45.557 | [M+H]⁺ | 295.133 | 294.12572 | 0.43 | C₁₉H₁₈O₃ | Tanshinone IIA* | 1764441430 |
| 109 | 46.173 | [M+H]⁺ | 401.19608 | 400.18877 | 0.46 | C₂₃H₂₈O₆ | Schisandrin B* | 30311185202 |
| 110 | 46.565 | [M+H]⁺ | 385.16467 | 384.1574 | 0.28 | C₂₂H₂₄O₆ | Schisandrin C | 1008788377 |
| 111 | 47.173 | [M+H]⁺ | 279.23215 | 278.22487 | 1.03 | C₁₈H₃₀O₂ | α-Linolenic acid | 296650304.8 |
| 112 | 47.562 | [M+H]⁺ | 307.26334 | 306.25606 | 0.59 | C₂₀H₃₄O₂ | Linolenic acid ethyl ester | 65951830.54 |

*** Indicates comparison with reference standards.**
