# Supplementary material for: Fuzheng Huayu tablets reduces the risk of further decompensation after the first decompensation in patients with HBV-related cirrhosis: protocol for a randomized, double-blind, placebo-controlled, multicenter trial
Source: Front Pharmacol. 2026 Jul 2;17:1828944. doi: 10.3389/fphar.2026.1828944 (PMC13373875; doi:10.3389/fphar.2026.1828944)
Supplement: Supplementary file 6 [file Supplementaryfile3.docx]

**Quantification of of marker compounds of Fuzheng Huayu**

**Materials and methods**

The quality control of Fuzheng Huayu was performed using a high-performance liquid chromatography fingerprint method for Fuzheng Huayu composition. Briefly, 1.00 g of Fuzheng Huayu sample powder was accurately weighed and transferred into a 10 mL volumetric flask. Approximately 5–8 mL of 50% methanol was added, and the sample was extracted by ultrasonication for 20 min. After extraction, the solution was diluted to volume with 50% methanol, mixed thoroughly, and filtered through a 0.45 μm microporous membrane. The subsequent filtrate was collected as the test solution for HPLC fingerprint analysis. Chromatographic separation was performed on an octadecylsilane-bonded silica gel C18 column. The mobile phase consisted of 0.05% phosphoric acid in water as solvent A and acetonitrile as solvent B. Gradient elution was applied with solvent A decreasing from 95% to 60% and then to 10%, and solvent B increasing from 5% to 40% and then to 90% over 120 min. The flow rate was maintained at 0.8–1.2 mL/min. The column temperature was controlled at 20–40°C, and the injection volume was 10–20 μL. The chromatograms were recorded at 280 nm, 254 nm and 261 nm.

The standard HPLC fingerprint was established using 10 independent batches of Fuzheng Huayu. Chromatographic peaks detected in different batches were compared, and common peaks were identified according to their retention times and ultraviolet absorption characteristics. The similarity between the sample fingerprint and the standard fingerprint was calculated using chromatographic fingerprint similarity evaluation software. A batch was considered qualified when the similarity value was not less than 0.80 (patent: CN101879229A).

**Results**

**1. Methodological evaluation**

**1.1 Specificity test**

The specificity of the HPLC fingerprint method was evaluated by comparing the chromatograms of Fuzheng Huayu samples with those of reference compounds and blank solvent. The major chromatographic peaks in the sample solution were well separated under the optimized chromatographic conditions, and no obvious interference from the solvent was observed. The results indicated that the method had acceptable specificity for the fingerprint-based quality assessment of Fuzheng Huayu (Figure 1).


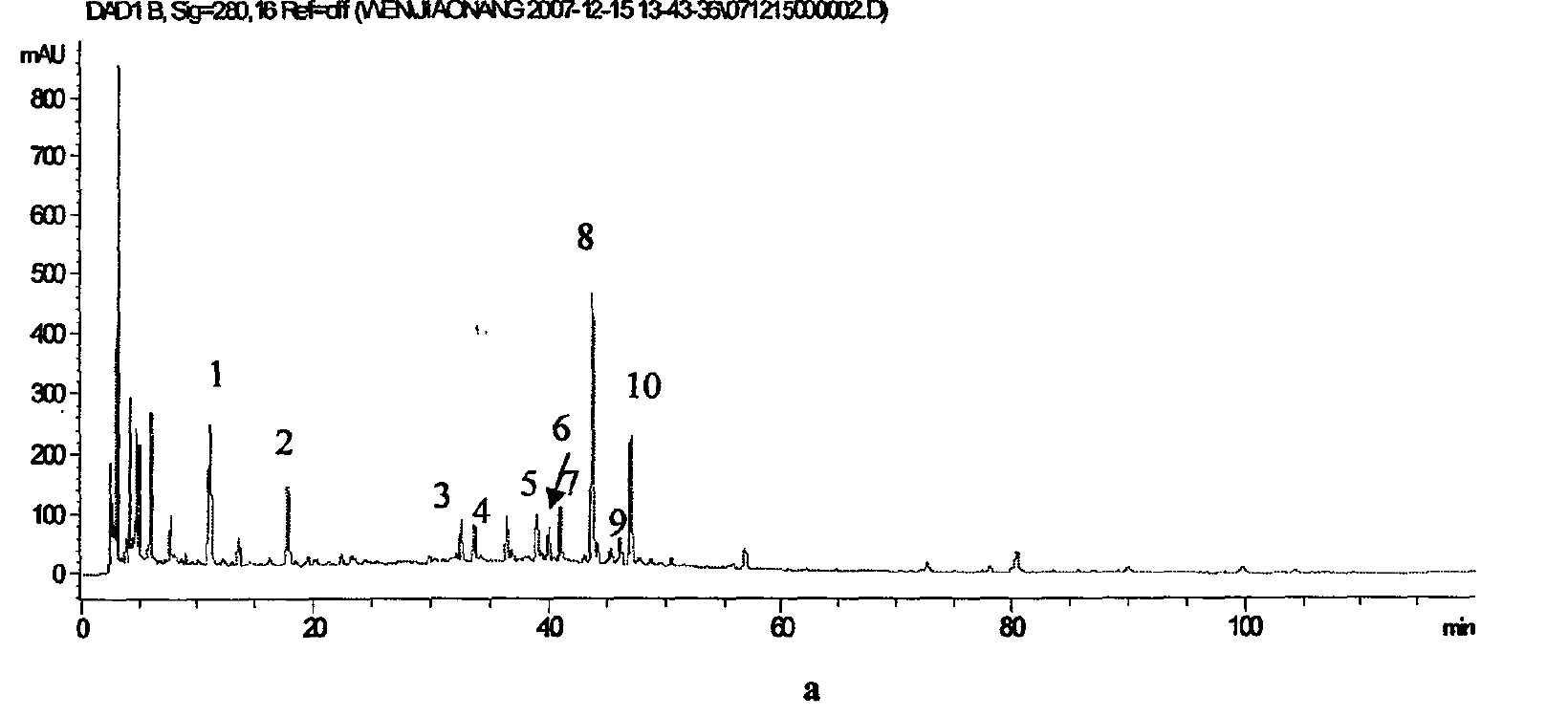


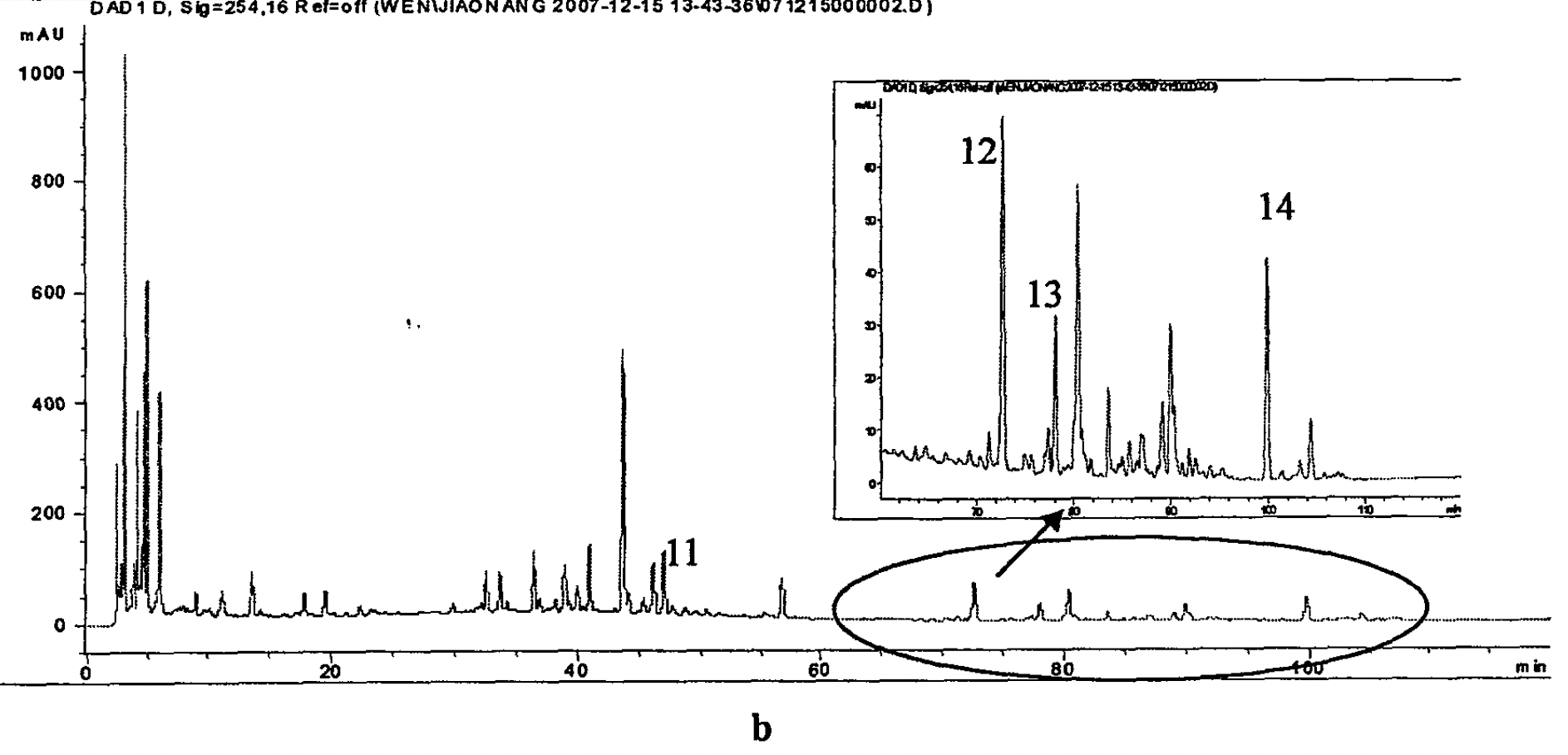


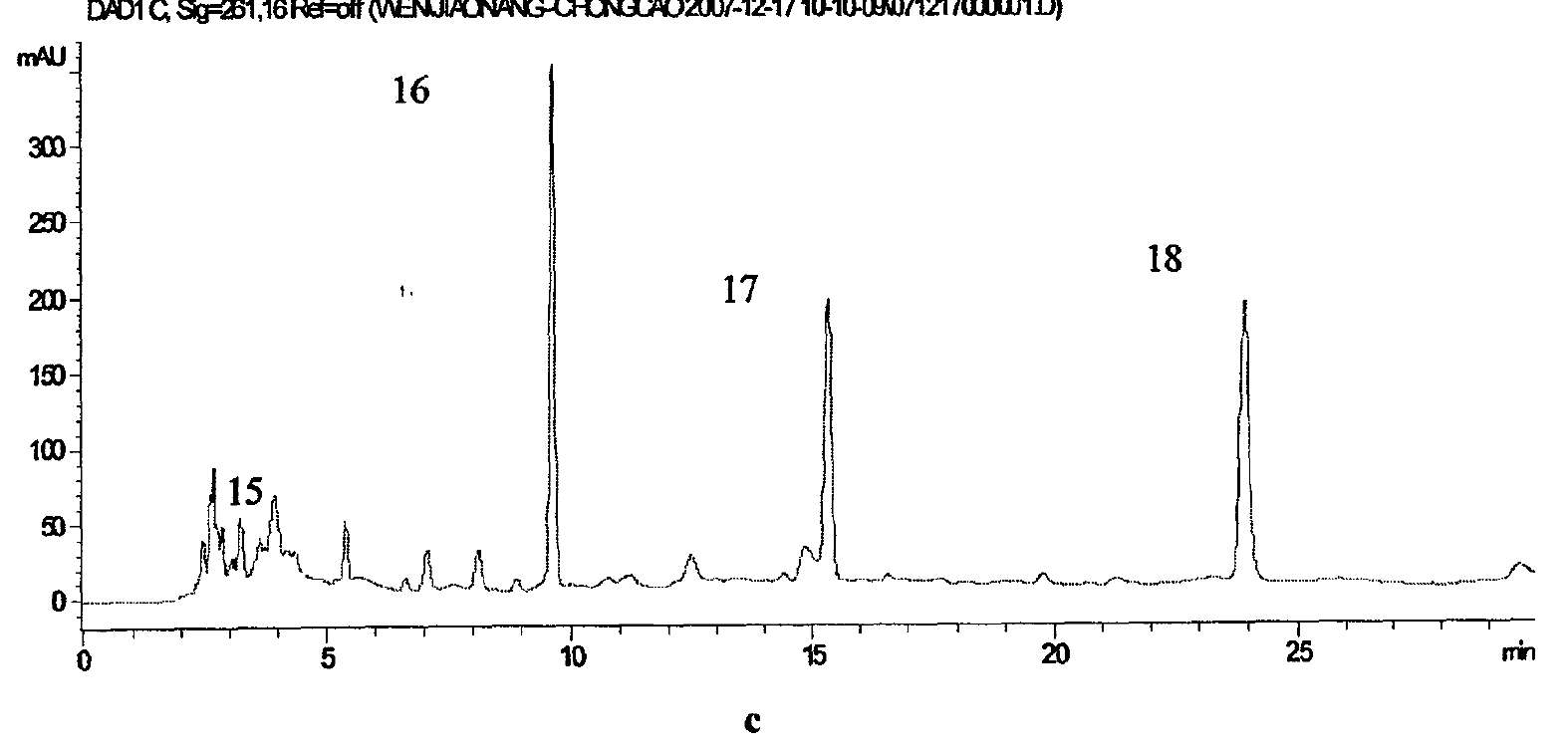


**Figure 1.** Representative HPLC fingerprint chromatograms of Fuzheng Huayu recorded at 280 nm (a), 254 nm (b), and 261nm (c). Peak assignment: 1, danshensu; 2, protocatechualdehyde; 5, rosmarinic acid; 8, salvianolic acid B; 12, schisandrol A; 13, schisandrol B; 14, schisandrin A; 16, Uridine, peak; 17, guanosine, peak; 18, adenosine.

**1.2 Repeatability**

Repeatability was evaluated by preparing multiple test solutions from the same batch of Fuzheng Huayu and analyzing them under identical chromatographic conditions. The retention times and peak areas of the major common peaks were compared. The chromatographic profiles were consistent among repeated preparations, indicating that the sample preparation and HPLC analysis method had acceptable repeatability.

**1.3 Precision**

Instrumental precision was assessed by repeated injection of the same Fuzheng Huayu test solution. The relative standard deviations of the retention times and peak areas of the common peaks were calculated. The results showed that the method had good instrumental precision and was suitable for fingerprint analysis of Fuzheng Huayu.

**1.4 Stability**

The stability of the test solution was evaluated by analyzing the same Fuzheng Huayu sample solution at different time points after preparation. The relative standard deviations of the retention times and peak areas of the common peaks were calculated. The chromatographic profiles remained stable during the tested period, indicating that the prepared sample solution was suitable for HPLC analysis within the validated time window.

**Sample analysis**

The established HPLC fingerprint method was applied to evaluate the quality of Fuzheng Huayu samples from different batches. Each batch was analyzed at 280 nm, 254 nm and 261nm. The chromatographic profiles of different batches were compared with the standard fingerprint, and similarity values were calculated.

The results showed that qualified Fuzheng Huayu samples had consistent chromatographic profiles, with the characteristic common peaks clearly detected at both wavelengths. The similarity values of qualified batches were not less than 0.80, indicating acceptable batch-to-batch consistency. These findings support the applicability of the HPLC fingerprint method for the identity confirmation and quality control of Fuzheng Huayu.
