# Supplementary material for: Fuzheng Huayu tablets reduces the risk of further decompensation after the first decompensation in patients with HBV-related cirrhosis: protocol for a randomized, double-blind, placebo-controlled, multicenter trial
Source: Front Pharmacol. 2026 Jul 2;17:1828944. doi: 10.3389/fphar.2026.1828944 (PMC13373875; doi:10.3389/fphar.2026.1828944)
Supplement: Supplementary file 7 [file Supplementaryfile4.docx]

**HPLC fingerprint of Fuzheng Huayu**

**Materials and methods**

Fuzheng Huayu samples were subjected to high-performance liquid chromatography fingerprint analysis for quality control. The chromatographic separation was performed using an octadecylsilane-bonded silica gel C18 column. The mobile phase consisted of 0.05% phosphoric acid aqueous solution as solvent A and acetonitrile as solvent B. Gradient elution was applied for chromatographic separation. The detection wavelength was set at 210 nm. The column temperature was maintained at 30°C, and the flow rate was set at 0.3 mL/min.

The system suitability was evaluated before sample analysis. The number of theoretical plates calculated based on the salvianolic acid B peak was required to be not less than 200,000. In addition, the resolution between the naringenin peak and the genistein peak was required to be greater than 1.0.

For preparation of the test solution, an appropriate amount of Fuzheng Huayu sample was accurately weighed and extracted with 50% methanol by ultrasonication. The extract was centrifuged, and the supernatant was collected for HPLC analysis.

The HPLC fingerprint of Fuzheng Huayu was established by comparing the retention time and peak pattern of characteristic chromatographic peaks. A reference fingerprint was generated and used for batch-to-batch consistency evaluation. The similarity evaluation system for chromatographic fingerprint analysis of traditional Chinese medicine was used to assess the similarity between test samples and the reference fingerprint.

**Results**

The HPLC fingerprint of Fuzheng Huayu was established under the optimized chromatographic conditions. A total of 18 characteristic peaks were identified in the reference fingerprint. These peaks represented chemical constituents derived from the major herbal components of Fuzheng Huayu, including Salvia miltiorrhiza, fermented Cordyceps sinensis mycelia powder, Persicae Semen, pine pollen, Gynostemma pentaphyllum, and Schisandra chinensis.

The characteristic peaks were assigned as follows: peak 1, adenosine; peak 2, guanosine; peak 3, danshensu; peak 4, protocatechualdehyde; peak 5, D-amygdalin; peak 6, rutin; peak 7, salvianolic acid D; peak 8, rosmarinic acid; peak 9, lithospermic acid; peak 10, salvianolic acid B; peak 11, salvianolic acid A plus kaempferol-3-O-rutinoside; peak 12, naringenin; peak 13, genistein; peak 14, massoia lactone; peak 15, schisandrol A; peak 16, schisandrol B; peak 17, schisandrin A; and peak 18, schisandrin B plus gomisin N.

Among these characteristic peaks, compounds derived from Salvia miltiorrhiza, including danshensu, salvianolic acid D, rosmarinic acid, lithospermic acid, salvianolic acid B, and salvianolic acid A, constituted the major phenolic acid-related fingerprint peaks. Schisandrol A, schisandrol B, schisandrin A, and schisandrin B plus gomisin N were mainly derived from Schisandra chinensis. D-amygdalin was derived from Persicae Semen, whereas rutin was mainly associated with Gynostemma pentaphyllum and Schisandra chinensis. Naringenin, genistein, and massoia lactone represented additional characteristic components derived from pine pollen or fermented Cordyceps sinensis mycelia powder.

The HPLC fingerprint showed a stable and characteristic chromatographic pattern for Fuzheng Huayu. The reference fingerprint contained well-resolved peaks with acceptable system suitability. The salvianolic acid B peak was selected as the reference peak for system suitability assessment because of its high abundance and representative contribution to the fingerprint profile. The results indicated that the established HPLC fingerprint method was suitable for the identification and quality consistency assessment of Fuzheng Huayu.

**Figure 1. HPLC fingerprint chromatogram of Fuzheng Huayu at 210 nm.**
The numbered peaks represent the characteristic constituents of Fuzheng Huayu. Peak 1, adenosine; peak 2, guanosine; peak 3, danshensu; peak 4, protocatechualdehyde; peak 5, D-amygdalin; peak 6, rutin; peak 7, salvianolic acid D; peak 8, rosmarinic acid; peak 9, lithospermic acid; peak 10, salvianolic acid B; peak 11, salvianolic acid A plus kaempferol-3-O-rutinoside; peak 12, naringenin; peak 13, genistein; peak 14, massoia lactone; peak 15, schisandrol A; peak 16, schisandrol B; peak 17, schisandrin A; and peak 18, schisandrin B plus gomisin N.
