# Supplementary material for: Fuzheng Huayu tablets reduces the risk of further decompensation after the first decompensation in patients with HBV-related cirrhosis: protocol for a randomized, double-blind, placebo-controlled, multicenter trial
Source: Front Pharmacol. 2026 Jul 2;17:1828944. doi: 10.3389/fphar.2026.1828944 (PMC13373875; doi:10.3389/fphar.2026.1828944)
Supplement: Supplementary file 8 [file Supplementaryfile5.doc]

**扶正化瘀片降低乙肝肝硬化首次失代偿患者发生再次失代偿事件：一项随机、双盲、安慰剂对照、多中心研究**

**知情同意书告知页**

**尊敬的患者：**

我们邀请您参加首都医科大学附属北京地坛医院开展的《扶正化瘀降低乙肝肝硬化首次失代偿患者发生再次失代偿事件：一项随机、双盲、安慰剂对照、多中心研究》课题研究。本研究将以首都医科大学附属北京地坛医院牵头，在全国多中心医院开展，估计将有432名受试者自愿参加。本知情同意书提供给您一些信息以帮助您确定是否参加此项研究，请您用一定的时间仔细阅读下面的内容，如有不清楚的问题或术语，可以与有关医师进行讨论。

您参加本项研究是完全自愿的。本次研究已经得到首都医科大学附属北京地坛医院伦理委员会的审查和批准。

本文涵盖的部分内容由法规要求而定，并且为了保护参加研究的患者的权益，本研究经伦理委员会审核并同意。

**1.为什么要开展本项研究？**

本课题拟开展一项随机、双盲、安慰剂对照、多中心研究，将432例符合入排标准的乙肝肝硬化首次失代偿患者纳入研究。对照组给予安慰剂联合常规西医抗病毒治疗，治疗组给予扶正化瘀片联合常规西医抗病毒治疗，治疗48周，从治疗开始即随访，共随访96周。以48周再次失代偿事件累积发生率为主要评价指标，结合肝纤维化指标、病毒学、影像学、中医证候积分、心电图、免疫细胞亚群等指标，观察扶正化瘀片降低乙肝肝硬化首次失代偿患者发生再次失代偿事件的疗效及安全性，为临床实践提供依据。

**2.该研究是怎样进行的？**

本研究为双盲，在研究期间，研究者和受试者均不知道分组情况，采用了1:1随机分组的方法，在您或您家人完全知情并书面同意的情况下，您不能自主选择自己的组别，您将会被随机分到安慰剂组或扶正化瘀片组，并完善相关实验室检查。无论您随机分到哪一组，您所接受的治疗措施都是目前治疗中常规使用的治疗方案。疗程为48周，我们会定期对您进行相关的检查和随访。治疗及随访期间，我们愿意为您提供持续的治疗指导。

安慰剂组：在剂型、形状、颜色、质地、气味、用法用量，与试验药物完全一致，但不含药物的活性成分，每天3次，每次4片（0.4g）治疗48周。联合常规西医治疗：对症支持治疗+抗病毒治疗：推荐恩替卡韦（ETV）/替诺福韦酯（TDF）/丙酚替诺福韦（TAF）/艾米替诺福韦（TMF）。

扶正化瘀组：扶正化瘀片组成包含丹参、桃仁、绞股蓝、松花粉、冬虫夏草、五味子，具有活血化瘀、养肝益精功效。扶正化瘀每天3次，每次4片（0.4g）治疗48周。联合常规西医治疗：对症支持治疗+抗病毒治疗：推荐恩替卡韦（ETV）/替诺福韦酯（TDF）/丙酚替诺福韦（TAF）/艾米替诺福韦（TMF）。

**3.本研究是否涉及血液等标本的采集？**

本研究涉及静脉血标本的保存、检测，且该静脉血标本分析仅限于本受试者知情同意书描述之内的用途研究，不会用于其他目的；

您是否自愿同意将本研究剩余的生物样本进行捐献，并存入医院样本库中，以用于未来其他研究。是否同意，请您在签字页上选择确认。

**4.参加本研究的条件?**

入选标准：自愿入组，能理解和签署知情同意书；年龄18-80岁，性别不限；筛选时HBsAg阳性≥6个月；符合中医证型：瘀血阻络，肝肾不足证，症见胁下痞块，胁肋疼痛，面色晦暗，或见赤缕红斑，腰膝酸软，疲倦乏力，头晕目涩，舌质暗红或有瘀斑，苔薄或微黄，脉弦细；发生首次失代偿事件，即符合《肝硬化诊治指南(2019年版)》失代偿期肝硬化诊断标准：(1)具备肝硬化的诊断依据；(2)出现门静脉高压相关并发症:如腹水、食管胃静脉曲张破裂出血、肝性脑病、肝肾综合征等。

排除标准：合并甲肝、丙肝、丁肝、戊肝和/或HIV感染；合并自身免疫性肝病、酒精性肝病、药物性肝病等其他肝脏疾病；合并恶性肿瘤者；有脾切除手术史，经颈静脉肝内门体分流术史；神经精神疾病者，尤其是抑郁、焦虑、躁狂、精神分裂症等精神疾病史或有精神疾病家族史（尤其是抑郁症疾病史或抑郁倾向者）；合并严重的心脏、肺、肾脏等器官障碍者；计划接受器官移植或已经进行过器官移植者；妊娠、哺乳期妇女或在研究期间有生育计划者；对扶正化瘀、核苷（酸）类似物或药物过敏者，或符合试验用药品说明书中任何一条禁忌症者；6个月内曾服用扶正化瘀及其他抗肝纤维化中药饮片/中成药者；筛选前3个月内参加过其他干预性试验研究或研究者认为不适宜入组的其他情况。

**5.研究中我的责任是什么？**

为了能使本项研究顺利和成功开展，请遵守以下规定：

- 您需要遵照研究者安排治疗、接受检查；
- 在与研究医生确认之前，您不能随意改变您目前的治疗或者开始任何新的治疗；
- 您需要告知研究医生有关您健康的问题，甚至是您认为不是很重要的问题；
- 您需要告知研究医生您在参加研究前和研究中使用的除研究治疗方案（用药或其他）以外的其他所有治疗手段（包含中草药）；
- 在整个研究期间您不能再参加其他任何有关药物或者医疗器械的临床研究；
- 在研究期间会在治疗前第0周，第12周，第24周，第36周，第48周，第72周，第96周从您的胳膊上每次抽取不多于20ml的静脉血，共7次。其中，20ml静脉血包括常规复查15ml（肝肾功能、甲胎蛋白、凝血功能、血常规、血清病毒学）和科研研究样本采集5ml(血浆和免疫细胞检测)。同时，每月对您进行相关病情访问，此外您需要告诉我们您的任何变化。

**6.参加研究的风险与不适有哪些？**

研究过程中您可能会出现不良反应，但不包括疾病本身恶化，研究过程中您可能会出现的风险包括：

- 中药及安慰剂服用风险：过敏反应，如皮肤瘙痒、红疹；及胃肠道症状，如腹泻。
- 检查风险：包括采血不适，如采血时可能会导致皮肤瘀斑，极少数情况下，针头刺破部位皮肤可能会发生感染。

我们会监测研究中所有病人的任何不良反应。如果您在访视之间出现任何不良反应，请及时给您的研究医生打电话咨询。

**7.从此研究中我能得到什么受益？**

参加本研究可能会、也可能不会使您的健康状况好转。从本研究中得到的信息将有助于对与您病情相同的病人有指导意义。

**8.我是否有其他的治疗选择？**

参加本研究可能改善或不能改善您的健康状况，您可以选择：

- 不参加本研究，继续您的常规治疗：如抗炎保肝等对症治疗、抗病毒治疗等。
- 参加其他研究。

请与您的医生协商您的决定。

**9.参加本研究会给予我什么报酬及相关费用？**

研究过程中，在筛选期间支付受试者交通及营养补助600元/人，符合入组后每完成一次随访支付受试者交通及营养补助500元/人，共6次随访，合计3600元/人。您参与本研究期间服用的扶正化瘀片及安慰剂药物为免费提供，本研究期间所做的临床常规化验检查费用（包括传染病筛查、血常规、尿常规、肝肾功能、肝纤维化、乙肝病毒学、凝血功能、甲状腺功能、甲胎蛋白、腹部超声、肝脏弹性、腹部MR/CT、对于消化道出血的需做胃镜）需您自己承担，用于实验室科研检测（血浆及免疫细胞检测）费用为免费。为了补偿您参加本研究可能给您带来的不便，我们将对患者随时提供病情相关医学专业知识指导，希望您能坚持完成本观察。

**10.如果我在参加研究期间受到损害会怎样？**

如果您的健康确因参加这项研究而发生与研究相关的损害，请立即通知研究医生，他们将负责对您采取积极的治疗措施。

即使您已经签署这份知情同意书，您仍然保留您所有的合法权利。

**11.我必须参加研究吗？**

参加本研究是完全自愿的，您可以拒绝参加研究，或者研究过程中的任何时候选择退出，不需要任何理由。该决定不会影响您未来的治疗，您的任何医疗待遇与权益不会因此而受到影响。如果您决定退出本研究，请提前通知您的研究医生。为了保障您的安全，您可能被要求进行相关检查，这对保护您的健康是有利的。

一旦您决定参加本项研究，请您签署此知情同意书表明同意，进入研究前，研究医生会为您做筛查以确认是否为合适人选。

**12.我的个人信息是保密的吗？**

在研究期间，您的姓名、性别等个人资料将用代号或数字代替，并予以严格的保密，只有相关的医生知道您的资料，您的隐私权会得到很好的保护，研究结果可能会在杂志上发表，但不会泄露您个人的任何资料。

如果您同意参加本项研究，您所有的医疗资料都将被开展研究的医院，研究者、研究主管部门、伦理委员会查阅，以检查研究的操作是否恰当，如果您签署了知情同意书，也就意味着您同意接受上述人员的查阅。

**13.研究中如何获得帮助？**

您可随时了解与本研究相关的信息资料和研究进展，如果您有与本研究相关的任何问题，请联系您的医生 ，电话 010-84322313 。

如果您有与自身权利/权益相关的任何问题，或者您想反映参与本研究过程中遭遇的困难、不满和忧虑，或者想提供与本研究有关的意见和建议，请联系北京地坛医院伦理委员会（电话：010-84322127）。

**知情同意书签字页**

**受试者同意声明**

如果您完全理解这一项研究项目的内容，并同意参加此项研究，您将签署此知情同意书，一式两份，由研究者和受试者本人或委托人各保留一份。

临床研究项目名称：扶正化瘀降低乙肝肝硬化首次失代偿患者发生再次失代偿事件：一项随机、双盲、安慰剂对照、多中心研究

**关于参加本项临床试验的同意声明：**

1. 我已阅读并理解此项研究的知情同意书，且研究人员已经对研究过程中可能出现的问题及解决方案向我进行解释说明；
2. 我已经讨论并询问了有关本研究的相关问题，这些问题的解答令我满意；
3. 我有充足的时间作出决定；
4. 我是自愿同意参加本文所介绍的临床研究，拒绝参加研究不会损害我应有的任何利益；
5. 我已经获知在研究中我该咨询的研究人员名单；
6. 如本知情同意书所介绍，我同意研究人员和其他相关人员能够接触到我的医疗和个人信息；
7. 我同意研究医生可以告诉我的医生我正在参加一项研究。

签 名： 日 期：

姓名正楷： 联系电话：

法定代表人签名（如适用）： 日 期：

法定代表人姓名正楷： 联系电话：

见证人签名（如适用）： 日 期：

见证人姓名正楷： 联系电话：

研究者签名： 日 期：

研究者姓名正楷： 联系电话：

**关于捐献生物样本的同意声明（可自愿选择同意或是拒绝）：若选择“不同意”，此处无需签字**

- □我自愿同意将本人在本次研究中被采集的血液、尿液、粪便、组织等研究剩余标本捐献给医院生物样本库，用于支持其他符合国家法律规范的匿名化的科学研究，未来开展研究的伦理性由医院伦理委员会进行监督和审查。
- □不同意

签 名： 日 期：

姓名正楷： 联系电话：

**关于病历等信息用于未来研究的同意声明（可自愿选择同意或是拒绝）：若选择“不同意”，此处无需签字**

- □我自愿同意将本人在本次研究中被收集的病历等信息用于今后开展其他符合国家法律规范的匿名化的科学研究，未来开展研究的伦理性由医院伦理委员会进行监督和审查。
- □不同意

签 名： 日 期：

姓名正楷： 联系电话：
