# Supplementary material for: Fuzheng Huayu tablets reduces the risk of further decompensation after the first decompensation in patients with HBV-related cirrhosis: protocol for a randomized, double-blind, placebo-controlled, multicenter trial
Source: Front Pharmacol. 2026 Jul 2;17:1828944. doi: 10.3389/fphar.2026.1828944 (PMC13373875; doi:10.3389/fphar.2026.1828944)
Supplement: Supplementary file 9 [file Supplementaryfile6.doc]

Fuzheng Huayu Tablets reduces the risk of further decompensation after the first decompensation in patients with HBV-related cirrhosis: protocol for a randomized, double-blind, placebo-controlled, multicenter trial

Informed Consent Form - Information Page

Dear Patient,

You are invited to participate in the clinical study entitled Fuzheng Huayu Tablets reduces the risk of further decompensation after the first decompensation in patients with HBV-related cirrhosis: protocol for a randomized, double-blind, placebo-controlled, multicenter trial, sponsored by Beijing Ditan Hospital Affiliated to Capital Medical University. This multicenter study will be led by Beijing Ditan Hospital Affiliated to Capital Medical University and conducted in multiple hospitals nationwide, with an estimated 432 eligible subjects participating on a voluntary basis. This informed consent form provides you with key information to help you decide whether to take part in the study. Please take time to read the content carefully, and you may discuss any unclear questions or terms with the investigating physicians.

Your participation in this study is completely voluntary. The study has been reviewed and approved by the Ethics Committee of Beijing Ditan Hospital Affiliated to Capital Medical University.

Certain content in this form is required by regulatory guidelines. To protect the rights and interests of study participants, the study has been examined and approved by the Ethics Committee.

1. Rationale for the Study

This randomized, double-blind, placebo-controlled, multicenter study will enroll 432 patients with HBV-related cirrhosis who have experienced their first decompensation event and meet the inclusion and exclusion criteria. The control group will receive placebo combined with conventional Western medical antiviral therapy, while the treatment group will receive Fuzheng Huayu Tablets combined with conventional Western medical antiviral therapy for a 48-week treatment period, with follow-up starting at the initiation of treatment and lasting for a total of 96 weeks. The primary outcome measure is the cumulative incidence of recurrent decompensation events at 48 weeks. Secondary outcome measures include liver fibrosis markers, virological indices, imaging findings, TCM syndrome scores, electrocardiogram results, and immune cell subsets. The study aims to evaluate the efficacy and safety of Fuzheng Huayu Tablets in reducing further decompensation events in this patient population and to provide evidence for clinical practice.

2. Study Procedures

This is a double-blind study, meaning neither the investigators nor the subjects will know the group assignments during the study period. Subjects will be randomly assigned to either the placebo group or the Fuzheng Huayu Tablets group at a 1:1 ratio. Upon full informed written consent by you or your family member, you will not be able to choose your study group. Relevant laboratory tests will be completed after randomization. Regardless of the group you are assigned to, the treatment you receive will be a routine clinical regimen. The treatment course is 48 weeks, and you will undergo regular examinations and follow-up visits. Continuous treatment guidance will be provided during the treatment and follow-up periods.

Placebo Group: Placebo with the same dosage form, shape, color, texture, odor, and administration schedule as the study drug (no active pharmaceutical ingredients), 4 tablets (0.4g each) three times a day for 48 weeks, combined with conventional Western medical therapy including symptomatic supportive care and antiviral therapy (recommended: Entecavir (ETV) / Tenofovir Disoproxil Fumarate (TDF) / Tenofovir Alafenamide (TAF) / Amtenofovir (TMF)).

Fuzheng Huayu Group: Fuzheng Huayu Tablets contain Salvia miltiorrhiza Bunge [Lamiaceae; Salviae miltiorrhizae radix et rhizoma], Cordyceps sinensis (Berk.) Sacc. [Ophiocordycipitaceae; Cordyceps], Schisandra chinensis (Turcz.) Baill. [Schisandraceae; Schisandrae Chinensis Fructus], Gynostemma pentaphyllum (Thunb.) Makino [Cucurbitaceae; Gynostemmatis Herba], Pinus massoniana Lamb. [Pinaceae; Pini Pollen], and Prunus persica (L.) Batsch [Rosaceae; Persicae Semen], with the effects of promoting blood circulation to remove blood stasis and nourishing the liver and essence. The dosage is 4 tablets (0.4g each) three times a day for 48 weeks, combined with conventional Western medical therapy including symptomatic supportive care and antiviral therapy (recommended: Entecavir (ETV) / Tenofovir Disoproxil Fumarate (TDF) / Tenofovir Alafenamide (TAF) / Amtenofovir (TMF)).

3. Collection of Biological Samples (e.g., Blood)

Venous blood samples will be collected, stored and tested for the sole purpose of the research described in this informed consent form, and will not be used for any other objectives.

You may voluntarily choose to donate the remaining biological samples from this study to the hospital biobank for future research. Please confirm your choice on the signature page of this form.

4. Eligibility Criteria for Study Participation

Inclusion Criteria：Voluntarily enroll and able to understand and sign the informed consent form; aged 18 to 80 years, of either gender; positive HBsAg for ≥ 6 months at screening;Conforms to the TCM syndrome type of blood stasis obstructing the collaterals and liver-kidney deficiency, with symptoms including hypochondriac mass, hypochondriac pain, dark facial complexion, or spider nevi and erythema, soreness and weakness of the waist and knees, fatigue and lassitude, dizziness and dry eyes, dark red tongue or with ecchymoses, thin or slightly yellowish fur, and string-like and thready pulse; experienced the first decompensation event, meeting the diagnostic criteria for decompensated cirrhosis in the Guidelines for the Diagnosis and Treatment of Cirrhosis (2019 Edition): (1) With diagnostic evidence of cirrhosis; (2) Presenting with portal hypertension-related complications such as ascites, esophagogastric variceal bleeding, hepatic encephalopathy, hepatorenal syndrome, etc.

Exclusion Criteria：Co-infected with HAV, HCV, HDV, HEV and/or HIV;complicated with other liver diseases such as autoimmune liver disease, alcoholic liver disease, drug-induced liver injury; complicated with malignant tumors; a history of splenectomy or transjugular intrahepatic portosystemic shunt (TIPS); a history of neuropsychiatric diseases, especially depression, anxiety, mania, schizophrenia, or a family history of neuropsychiatric diseases (especially depression or depressive tendency);Complicated with severe cardiac, pulmonary, renal or other organ dysfunctions; planned to receive or have received organ transplantation; pregnant, lactating women or women with fertility plans during the study period; allergic to Fuzheng Huayu Tablets, nucleos(t)ide analogs or the study drugs, or meeting any contraindication in the study drug package insert; having taken Fuzheng Huayu Tablets or other Chinese herbal decoctions/patent medicines for anti-liver fibrosis within 6 months; having participated in other interventional clinical trials within 3 months before screening, or other circumstances deemed ineligible by the investigator.

5. Your Responsibilities as a Study Participant

To ensure the smooth and successful conduct of the study, please comply with the following requirements:

- Follow the treatment and examination arrangements by the investigators;
- Do not arbitrarily change your current treatment or initiate any new treatment without confirmation from the study physician;
- Inform the study physician of any health problems, even those you consider insignificant;
- Inform the study physician of all other treatment methods (including Chinese herbal medicines) used before and during the study, except for the study treatment regimen (medication or other);
- Do not participate in any other clinical studies involving drugs or medical devices during the entire study period;
- Venous blood samples (no more than 20ml each time) will be collected from your arm at Week 0 (baseline), Week 12, Week 24, Week 36, Week 48, Week 72 and Week 96 during the study (7 times in total). The 20ml venous blood sample includes 15ml for routine follow-up tests (liver and kidney function, alpha-fetoprotein, coagulation function, blood routine, serum virology) and 5ml for research purposes (plasma and immune cell detection). In addition, you will receive a monthly disease follow-up interview, and you must inform us of any changes in your condition.

6. Potential Risks and Discomforts of Study Participation

You may experience adverse reactions during the study (excluding the natural progression of the underlying disease). Potential risks include:

- Risks associated with taking Chinese medicine and placebo: allergic reactions such as skin pruritus and rash; and gastrointestinal symptoms such as diarrhea;
- Risks associated with examinations: discomfort from blood collection, such as skin ecchymosis at the puncture site; in rare cases, infection may occur at the needle puncture site.

All adverse reactions in study participants will be monitored. If you experience any adverse reactions between follow-up visits, please contact your study physician immediately for consultation.

7. Potential Benefits of Study Participation

Your health condition may or may not improve by participating in this study. The information obtained from this study will provide valuable guidance for the treatment of other patients with the same condition.

8. Alternative Treatment Options

Participation in this study may or may not improve your health condition. Your alternative options include:

- Decline participation and continue your conventional treatment such as anti-inflammatory and hepatoprotective symptomatic treatment, antiviral therapy, etc.;
- Participate in other clinical studies.

Please discuss your decision with your physician.

9. Compensation and Related Costs for Study Participation

A transportation and nutrition subsidy of RMB 600 per person will be provided during the screening period; a transportation and nutrition subsidy of RMB 500 per person will be provided for each completed follow-up visit after enrollment (6 follow-up visits in total), with a total subsidy of RMB 3,600 per person.Fuzheng Huayu Tablets and placebo provided during the study are free of charge. Costs for routine clinical laboratory tests during the study (including infectious disease screening, blood routine, urine routine, liver and kidney function, liver fibrosis markers, HBV virology, coagulation function, thyroid function, alpha-fetoprotein, abdominal ultrasound, liver elastography, abdominal MR/CT, and gastroscopy for patients with gastrointestinal bleeding) shall be borne by you. Costs for laboratory research tests (plasma and immune cell detection) are free of charge.

To compensate for any inconvenience caused by your participation in the study, we will provide professional medical guidance related to your condition at any time, and we hope you will persist in completing the study.

10. Compensation for Injury Sustained During Study Participation

If your health is harmed as a direct result of participating in this study, please notify the study physician immediately. Appropriate and active medical treatment will be provided at the expense of the study sponsor.

Even if you have signed this informed consent form, you retain all your legal rights.

11. Voluntary Participation

Your participation in this study is completely voluntary. You may refuse to participate or withdraw from the study at any time for any reason without penalty. This decision will not affect your future medical treatment, and your medical benefits and rights will not be compromised in any way. If you decide to withdraw from the study, please notify your study physician in advance. For your safety, you may be required to undergo relevant examinations, which are beneficial to protecting your health.

Once you decide to participate in the study, please sign this informed consent form to indicate your agreement. Before enrollment, the study physician will conduct screening to confirm your eligibility.

12. Confidentiality of Personal Information

During the study, your personal information such as name and gender will be replaced with a code or number and kept strictly confidential, with access limited only to relevant physicians. Your privacy will be fully protected. Study results may be published in academic journals, but no personal identifying information will be disclosed.

If you agree to participate in this study, all your medical records will be accessible to the conducting hospital, investigators, study supervision departments and the Ethics Committee to inspect the proper conduct of the study. By signing the informed consent form, you consent to such access.

13. How to Obtain Assistance During the Study

You may obtain information about the study and its progress at any time. If you have any questions related to the study, please contact your study physician at 010-84322313.

If you have any questions about your rights and interests as a study participant, or wish to report difficulties, dissatisfaction or concerns encountered during the study, or provide comments and suggestions related to the study, please contact the Ethics Committee of Beijing Ditan Hospital at 010-84322127.

Fuzheng Huayu Tablets reduces the risk of further decompensation after the first decompensation in patients with HBV-related cirrhosis: protocol for a randomized, double-blind, placebo-controlled, multicenter trial

Informed Consent Form - Signature Page

Subject's Consent Statement

If you fully understand the content of this study and agree to participate, you will sign this informed consent form (in duplicate), with one copy retained by the investigator and one copy by the subject or his/her authorized representative.

Clinical Study Title: Fuzheng Huayu Tablets reduces the risk of further decompensation after the first decompensation in patients with HBV-related cirrhosis: protocol for a randomized, double-blind, placebo-controlled, multicenter trial

Consent Statement for Participation in This Clinical Trial:

1. I have read and understood the informed consent form for this study, and the investigators have explained the potential problems and solutions during the study to me;

2. I have discussed and asked all relevant questions about the study, and I am satisfied with the answers;

3. I have been given sufficient time to make my decision;

4. I voluntarily agree to participate in the clinical study described in this form, and refusal to participate will not prejudice any of my legitimate interests;

5. I have been informed of the list of investigators to consult for the study;

6. As described in this informed consent form, I agree that the investigators and other relevant personnel may access my medical and personal information;

7. I agree that the study physician may inform my attending physician that I am participating in this study.

Signature: _________________________ Date: _________________________

Name (in block letters): _________________________ Contact Phone: _________________________

Legal Representative's Signature (if applicable): _________________________ Date: _________________________

Legal Representative's Name (in block letters): _________________________ Contact Phone: _________________________

Witness's Signature (if applicable): _________________________ Date: _________________________

Witness's Name (in block letters): _________________________ Contact Phone: _________________________

Investigator's Signature: _________________________ Date: _________________________

Investigator's Name (in block letters): _________________________ Contact Phone: _________________________

Consent Statement for Donation of Biological Samples (Voluntary Choice: Agree/Disagree)

No signature required if "Disagree" is selected

☐ I voluntarily agree to donate the remaining biological samples (e.g., blood, urine, feces, tissue) collected from me in this study to the hospital biobank for supporting other anonymous scientific research in compliance with national laws and regulations. The ethical review of future research will be supervised and examined by the hospital Ethics Committee.

☐ Disagree

Signature: _________________________ Date: _________________________

Name (in block letters): _________________________ Contact Phone: _________________________

Consent Statement for Use of Medical Records and Other Information in Future Research (Voluntary Choice: Agree/Disagree)

No signature required if "Disagree" is selected

☐ I voluntarily agree to the use of my medical records and other information collected in this study for future anonymous scientific research in compliance with national laws and regulations. The ethical review of future research will be supervised and examined by the hospital Ethics Committee.

☐ Disagree

Signature: _________________________ Date: _________________________

Name (in block letters): _________________________ Contact Phone: _________________________
